# Late gene expression–deficient cytomegalovirus vectors elicit conventional T cells that do not protect against SIV

**DOI:** 10.1172/jci.insight.164692

**Published:** 2023-03-22

**Authors:** Scott G. Hansen, Jennie L. Womack, Wilma Perez, Kimberli A. Schmidt, Emily Marshall, Ravi F. Iyer, Hillary Cleveland Rubeor, Claire E. Otero, Husam Taher, Nathan H. Vande Burgt, Richard Barfield, Kurt T. Randall, David Morrow, Colette M. Hughes, Andrea N. Selseth, Roxanne M. Gilbride, Julia C. Ford, Patrizia Caposio, Alice F. Tarantal, Cliburn Chan, Daniel Malouli, Peter A. Barry, Sallie R. Permar, Louis J. Picker, Klaus Früh

**Affiliations:** 1 Vaccine and Gene Therapy Institute and Oregon National Primate Research Center, Oregon Health & Science University, Beaverton, Oregon, USA.; 2 California National Primate Research Center, UCD, Davis, California, USA.; 3 Duke Human Vaccine Institute, Duke University Medical School, Durham, North Carolina, USA.; 4 Department of Pediatrics, Weill Cornell Medicine, New York, New York, USA.; 5 Department of Biostatistics and Bioinformatics, Duke University Medical Center, Durham, North Carolina, USA.; 6 Center for Human Systems Immunology, School of Medicine, Duke University, Durham, North Carolina, USA.; 7 Departments of Pediatrics and Cell Biology and Human Anatomy, School of Medicine, UCD, Davis, California, USA.

**Keywords:** AIDS/HIV, Virology, AIDS vaccine, T cells

## Abstract

Rhesus cytomegalovirus–based (RhCMV-based) vaccine vectors induce immune responses that protect ~60% of rhesus macaques (RMs) from SIV_mac239_ challenge. This efficacy depends on induction of effector memory–based (EM-biased) CD8^+^ T cells recognizing SIV peptides presented by major histocompatibility complex-E (MHC-E) instead of MHC-Ia. The phenotype, durability, and efficacy of RhCMV/SIV-elicited cellular immune responses were maintained when vector spread was severely reduced by deleting the antihost intrinsic immunity factor phosphoprotein 71 (pp71). Here, we examined the impact of an even more stringent attenuation strategy on vector-induced immune protection against SIV. Fusion of the FK506-binding protein (FKBP) degradation domain to Rh108, the orthologue of the essential human CMV (HCMV) late gene transcription factor UL79, generated RhCMV/SIV vectors that conditionally replicate only when the FK506 analog *Shield-1* is present. Despite lacking in vivo dissemination and reduced innate and B cell responses to vaccination, Rh108-deficient 68-1 RhCMV/SIV vectors elicited high-frequency, durable, EM-biased, SIV-specific T cell responses in RhCMV-seropositive RMs at doses of ≥ 1 *×* 10^6^ PFU. Strikingly, elicited CD8^+^ T cells exclusively targeted MHC-Ia–restricted epitopes and failed to protect against SIV_mac239_ challenge. Thus, Rh108-dependent late gene expression is required for both induction of MHC-E–restricted T cells and protection against SIV.

## Introduction

Cytomegalovirus-based (CMV-based) vaccines aim to exploit the unique immunological adaptations of this ubiquitous virus (1–3). These include host manipulation and immune evasion strategies that support viral persistence and efficient superinfection while maintaining robust antiviral immunity resulting in clinically inapparent infections in the majority of individuals (4, 5). Virus levels are extremely low in subclinical infections, yet both CD4^+^ and CD8^+^ T cell responses to human CMV (HCMV) antigens comprise, on average, ~10% of circulating memory T cells (6). These durable responses manifest a highly EM-biased T cell phenotype providing potent immediate effector function (7, 8). The original concept for CMV vectors proposed that durable prepositioning of high-frequency, EM-differentiated T cells (T_EM_) in tissues, particularly at portals of pathogen entry, would provide an effective intercept of pathogens without the inherent delay of an anamnestic effector response. Early intercept would control immune-evasive pathogens before they could fully implement their immune-evasion strategies (9). Indeed, using Rhesus cytomegalovirus–based (RhCMV-based) vectors in rhesus macaque (RM) models of HIV, tuberculosis, and malaria infection, we demonstrated that robust CD4^+^ and CD8^+^ T_EM_ can be elicited to essentially any heterologous pathogen insert in the setting of reinfection and that these responses mediate significant efficacy consistent with early pathogen intercept (10–13). Perhaps most compelling is the “control and clear” protection afforded by RhCMV/SIV vectors based on strain 68-1 against mucosal challenge with highly pathogenic SIV. Across multiple studies, 59% of RMs vaccinated with RhCMV/SIV demonstrate, without anamnestic expansion or anti-SIV antibodies, complete viral replication arrest 1–2 weeks after challenge, with the vast majority of protected animals clearing the initially established infection over the subsequent months to several years (10, 14–17)

Further analysis of RhCMV/SIV vector efficacy revealed another layer of complexity resulting from the unique immunobiology of CMV; CD8^+^ T cell responses elicited by strain 68-1 RhCMV vectors did not target epitopes presented by highly polymorphic MHC-Ia, but rather, epitopes were either restricted by MHC-II or by nonpolymorphic major histocompatibility complex-E (MHC-E) (18, 19). This unconventional epitope targeting was not observed in WT RhCMV infection but only in animals immunized with vectors derived from strain 68-1 (16, 18, 19). By repairing genetic alterations that spontaneously occurred in this strain during prolonged in vitro passaging, we generated a WT-like full-length genome (20). We demonstrated that 2 genomic regions need to be deleted to enable the induction of MHC-II– and MHC-E–restricted CD8^+^ T cells and that 8 viral proteins are capable of preventing the induction of unconventionally restricted CD8^+^ T cells. One region encodes 2 subunits (homologs of HCMV UL128 and UL130) of the pentameric complex (PC) regulating tissue tropism, whereas the second region encodes 6 viral chemokine homologs of the HCMV UL146 family (16). In addition, a viral MHC-E peptide ligand embedded in the signal sequences of Rh67 or the HCMV homologue UL40 is required to elicit MHC-E–restricted CD8^+^ T cells (21). Finally, we found that 68-1 RhCMV/SIV vectors engineered to be unable to replicate in specific cell types through inhibition by cell type–specific microRNAs resulted in vectors that manifest MHC-E–only (miR-126 restriction in endothelial cells), MHC-II–only (miR-142 restriction in myeloid cells), and MHC-Ia–only (miR-126 + miR-142 restriction) CD8^+^ T cell epitope targeting (17). Importantly, only RhCMV/SIV vectors with the ability to elicit MHC-E–restricted CD8^+^ T cells protected against SIV challenge (16, 17, 21). Of note, a requirement for unconventional CD8^+^ T cell responses has not been demonstrated for RhCMV vector efficacy against tuberculosis or malaria (12, 13), and it remains unexplored for protective immunity against CMV.

Translation from RhCMV in RMs to HCMV vectors in humans requires not only translating the immune factors responsible for efficacy, but also vaccine vectors sufficiently safe for widespread use. Although WT HCMV and RhCMV infections are asymptomatic in immune-competent individuals, both viruses can cause serious disease in settings of immunological deficiency, suppression, or immaturity — e.g. HIV/AIDS, transplantation, or fetal development (22). An optimal vector design would retain all assets of the platform, including superinfection, robust and durable T_EM_ immunogenicity, and CD8^+^ T cell response programming, but it would be sufficiently attenuated to abrogate the possibility of vector-mediated disease, even in the absence of a fully functional immune system. In the RhCMV model, we recently described vectors that displayed limited spread in vivo due to deletion of phosphoprotein 71 (pp71), which is essential for counteracting host intrinsic immunity during lytic infection (23, 24). Despite a dramatic reduction of viral dissemination and a complete absence of vector shedding in urine, pp71-deficient 68-1 RhCMV maintained lasting, unconventionally restricted CD8^+^ T cells that protected against SIV with similar efficiency as pp71-intact vectors (15, 23). Similarly designed pp71-deleted HCMV/HIV vectors demonstrated reduced dissemination and reactivation in humanized mouse models yet maintained the ability to elicit HIV-specific T cells in RMs (25).

The pp71-deleted vectors are substantially spread-impaired, but they are not, as shown here, spread-negative when directly inoculated into fetal macaques, raising the question of whether additional attenuation would retain the necessary immunogenicity characteristics. We therefore explored an even more stringent attenuation strategy. Specifically, a “single-cycle” vector that can only infect 1 round of target cells and, therefore, is spread-negative. As originally defined for herpes simplex virus–based (HSV-based) experimental cancer vaccines, single-cycle vectors can establish and maintain latency, but genetic defects prevent them from generating infectious progeny (26, 27). Various strategies to compensate for debilitating defects are employed when growing single-cycle vectors in vitro to enable large-scale vector production. These strategies include transcomplementation by providing the deleted essential protein in transfected cell lines (28) or functional complementation by eliminating the host target of a given viral protein, thus eliminating the need for it (25). Another approach is fusion of an essential viral protein to a tunable (in vitro reversible) degradation domain, and this results in expression of the essential gene product during in vitro production but not after in vivo administration (29). Taking this latter option, we engineered a 68-1 vector in which the protein encoded by RhCMV *Rh108* was fused to a rapidly degraded variant of the FK506-binding protein (FKBP). The FKBP-degradation domain is stabilized by the FK506 analog *Shield-1*, resulting in conditional stability of FKBP-fusion proteins (30, 31). Rh108 was chosen because the homologs UL79 of HCMV and M79 of murine CMV (MCMV) are transcription factors required for viral late gene expression and are, thus, essential for the generation of viral progeny but not for early gene expression and viral genome replication (32, 33). *Shield-1*–dependent growth in vitro was previously demonstrated for FKBP-UL79–expressing HCMV (32). Similarly, we observed that FKBP-Rh108 expressing RhCMV displayed reduced late gene expression and manifested no detectable spread in vitro and in vivo in the absence of *Shield-1*, consistent with a single-cycle phenotype. However, at doses above 1 ***×*** 10^6^ PFU, such single-cycle 68-1 RhCMV vectors elicited and maintained high-frequency T_EM_ responses that were similar in magnitude and differentiation phenotype to responses elicited by the parental vectors. Strikingly and unexpectedly, FKBP-Rh108 vectors elicited MHC-Ia, but not MHC-II– or MHC-E–restricted CD8^+^ T cells. Presumably due to the lack of MHC-E–restricted CD8^+^ T cell responses, these vectors did not protect against SIV. Our data, thus, suggest that late viral gene expression and/or residual in vivo spreading is required for the induction of protective CD8^+^ T cell responses. While these results rule out the use of this single-cycle vector design as an attenuation strategy for HCMV-based HIV vaccines, this strategy could still be useful in situations where lasting conventional, effector-differentiated MHC-Ia–restricted CD8^+^ T cells are required and sufficient for protection against infectious diseases or cancer, potentially including CMV itself, particularly for HCMV-negative or immunocompromised vaccine recipients.

## Results

### Construction of conditional single-cycle RhCMV.

**Figure 1:**
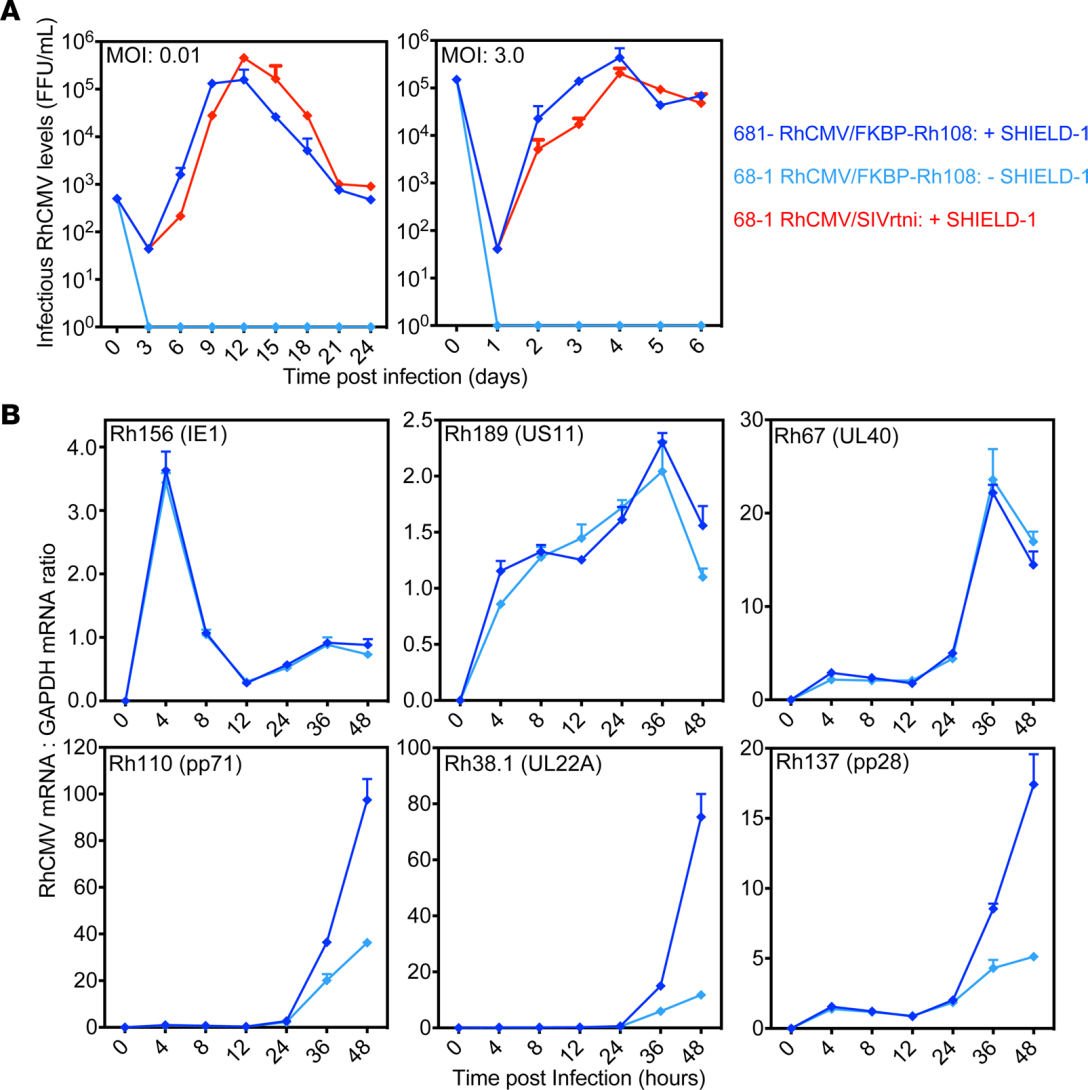
Conditional growth and late gene expression of RhCMV upon fusion of the FKBP destabilization domain to the UL79 homolog Rh108. (**A**) *Shield-1*–dependent growth of 68-1 RhCMV/ FKBP-Rh108/SIVrtni. Rhesus fibroblasts were infected either at a high or low multiplicity of infection (MOI) in the presence or absence of the FKBP-stabilizing compound *Shield-1* (30). For control, the parental vector 68-1 RhCMV/SIVrtni was used. Virus titers in cell culture supernatants collected at the indicated days were determined by immunofluorescence assay for pp65 expression in the presence or absence *of Shield-1*. The titers are shown as focus forming units (FFU) per mL of supernatant. Titers shown represent the arithmetic mean of 2 biological repeats titrated in triplicate, and data are shown as mean ± SEM. (**B**) *Shield-1*–dependent late gene expression by 68-1 RhCMV/FKBP-Rh108/SIVrtni. Rhesus fibroblasts were infected at an MOI of 3 in the presence or absence of *Shield-1*. Cells were harvested at the indicated times after infection, and mRNA levels of the indicated genes were determined in duplicate by qPCR. The results are shown as ratios compared with cellular GAPDH mRNA levels.

Using BAC recombineering, we fused the FKBP destabilization domain to the amino-terminus of Rh108 in 68-1 RhCMV containing an expression cassette for SIVrtni (a fusion of SIV Rev, Tat, Nef, and Integrase [Int]; refs. 10, 34) (Supplemental Figure 1A; supplemental material available online with this article; https://doi.org/10.1172/jci.insight.164692DS1). The resulting 68-1 RhCMV/FKBP-Rh108/SIVrtni recombinant was reconstituted in rhesus fibroblasts using *Shield-1* (Supplemental Figure 1B). Similar to FKBP-UL79–expressing HCMV (32), viral release into culture supernatants was dependent on *Shield-1* at both high and low multiplicity of infection (MOI) (Figure 1A). Consistent with a function of Rh108 as a transcription factor for viral late (L) genes without *Shield-1*, we observed reduced expression of viral mRNAs of the L genes *Rh38.1*, *Rh110* (pp71), and *Rh137* (pp28) but not the immediate early (IE) gene *Rh156* (IE1) or the early (E) genes *Rh189* or *Rh67* (Figure 1B). These genes are homologous to the HCMV *UL22A*, *UL82*, *UL99*, *UL123*, *US11*, and *UL40*, respectively (20, 35). In the presence of *Shield-1*, expression kinetics and levels of IE, E, and L genes were comparable with parental 68-1 (16). We conclude that fusion of the FKBP-degradation domain to the RhCMV homologue of HCMV UL79 results in *Shield-1*–dependent viral release from infected cells. The ability to maintain E gene expression required for viral genome replication while being deficient for expression of late genes encoding structural proteins required for viral assembly is consistent with a single-cycle vector in the absence of *Shield-1*.

### FKBP-Rh108 RhCMV does not spread in fetal RMs.

**Figure 2:**
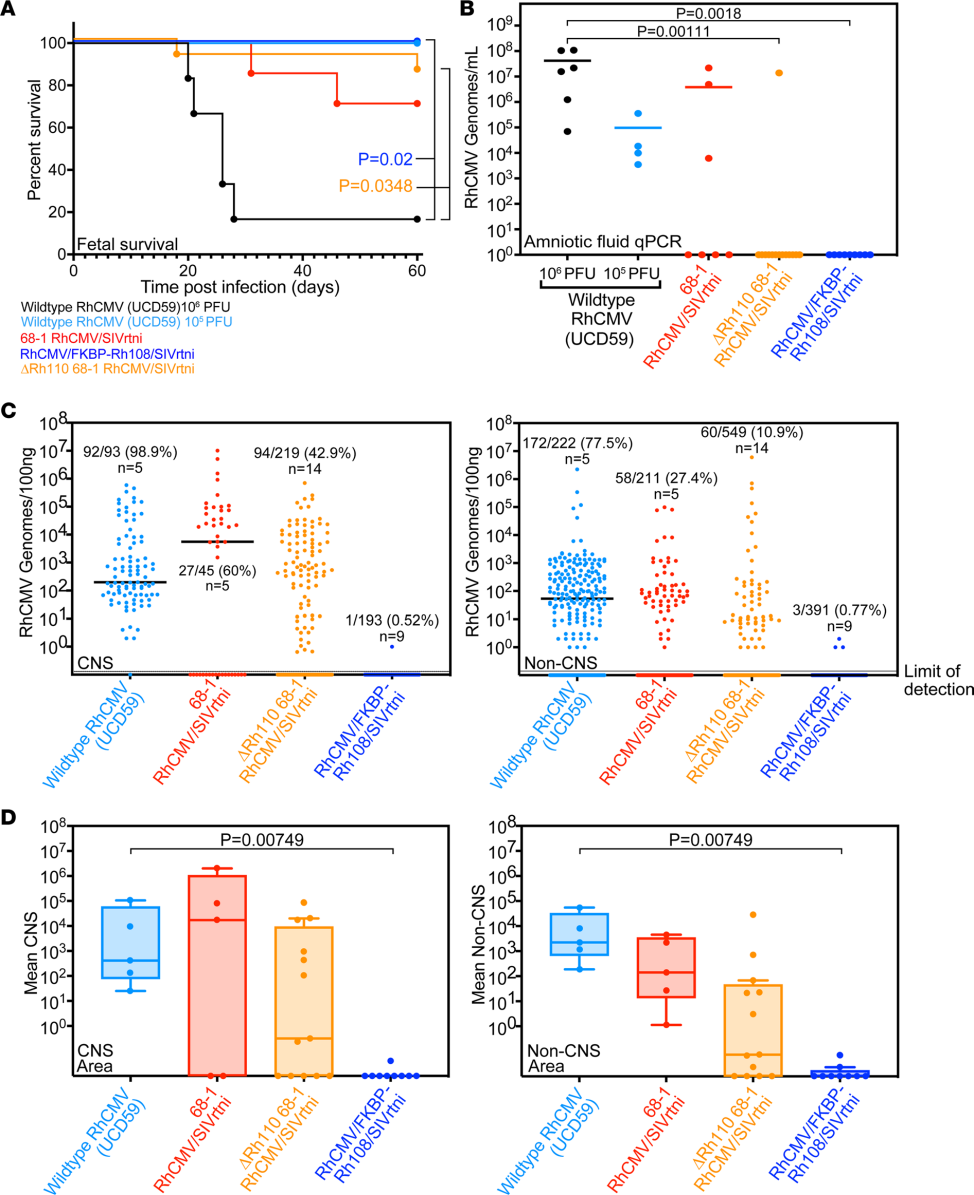
Rh108-deficiency abrogates RhCMV pathogenicity in fetal RMs. (**A**) The isolate UCD59 was inoculated either at 1 ***×*** 10^6^ PFU (*n* = 6) or at 1 ***×*** 10^5^ 1.6 ***×*** 10^5^ PFU (*n* = 4), whereas 68-1 RhCMV/SIVrtni (n = 7), 68-1 RhCMVΔRh110/SIVrtni (*n* = 14), or 68-1 RhCMV/FKPB-Rh108/SIVrtni (*n* = 9) were inoculated at 1 ***×*** 10^6^ PFU. The percentage of surviving fetuses is shown over time. Significant differences between the five groups were determined (log rank test, *P* < 0.0001), with post hoc pairwise comparison demonstrating that Rh108-FKBP and ΔRh110 differed significantly from the UCD59 1 ***×*** 10^6^ PFU cohort. Other comparisons were not significant (NS). (**B**) RhCMV genome copies per mL of amniotic fluid (AF) determined at the endpoint. Post hoc pairwise comparisons (after Kruskal Wallis test) were significant by Wilcoxon Rank Sum test with Holm’s multiple testing correction between the Rh108-FKBP or ΔRh110 cohorts and either UCD59 cohort (*P* value shown for 1 ***×*** 10^6^ PFU). (**C**) Genome copies determined in samples from surviving fetuses from CNS and non-CNS tissues (UCD59 represents one fetus from the 1 ***×*** 10^6^ and 4 fetuses from the 1 ***×*** 10^5^ group). Samples with above-threshold RhCMV detection are shown. Data for **C** are shown in Supplemental Figure 2, A and B. (**D**) Mean genome copies per fetus (except for sex-specific tissues; RM6.6; Supplemental Figure 2B) was not included due to limited tissue availability). There was a significant difference by Kruskal Wallis test between the four groups in both CNS (*P* = 0.00602) and non-CNS (*P* < 0.00001). Post hoc pairwise comparisons by Wilcoxon Rank Sum test suggested a significant difference between FKBP-Rh108 RhCMV and UCD59 (shown) or UCD59 together with 68-1 (*P* = 0.0138). ΔRh110-inoculated fetuses did not differ for CNS, whereas non-CNS samples differed from UCD59 (*P* = 0.0385) and UCD59 combined with 68-1 (*P* = 0.0266). Box plots show the median with whiskers extending to the farthest data point within 1.5***×*** interquartile range (IQR) above and below the box (first and third quartiles).

The lack of viral progeny without *Shield-1* suggested that virus would not spread in vivo, even in individuals with a compromised immune system, due to lack of the stabilizing ligand. Single-cycle MCMV was unable to spread in immunodeficient mice lacking the type I IFN receptor (36) or in SCID mice (37). To monitor spreading of Rh108-deficient RhCMV in an immunocompromised setting, we used direct RhCMV inoculation into fetal RMs, since early-gestation fetal RMs lack a fully functional adaptive immune system and are, thus, unable to control viral spread to all tissues, including the fetal brain (38, 39). We inoculated 9 fetuses in utero by i.p. injection under ultrasound guidance late in the first trimester of pregnancy with 1 ***×*** 10^6^ PFU of 68-1 RhCMV/FKBP-Rh108/SIVrtni. For controls, we used both the low-passage isolate UCD59 (39, 40) and the parental 68-1 RhCMV/SIVrtni vector, both of which were expected to manifest fetal disease at a dose of 1 ***×*** 10^6^ PFU (38). A total of 10 fetuses were administered UCD59 — 6 at 1 ***×*** 10^6^ PFU and 4 at 1 ***×*** 10^5^ to 1.6 ***×*** 10^5^ PFU (Supplemental Figure 2). Seven fetuses were inoculated with 1 ***×*** 10^6^ PFU of 68-1 RhCMV/SIVrtni. For additional comparison, we inoculated 14 fetuses with the pp71-deficient 68-1 RhCMVΔRh110/SIVrtni shown to be spread impaired in adult, immunocompetent RMs (23). RhCMVΔRh110 was either grown without complementation (*n* = 7) or recovered from complementing cells (*n* = 7), with the latter being potentially more infectious since pp71 is incorporated into virions (23). Five of the 6 fetuses inoculated with 1 ***×*** 10^6^ PFU of UCD59 were spontaneously aborted between 19 and 33 days postinoculation (dpi). In contrast, all fetuses inoculated with the lower doses of UCD59, 5 of 7 fetuses inoculated with 68-1 RhCMV/SIVrtni, 12 of 14 fetuses inoculated with 68-1 RhCMVΔRh11/SIVrtni, and all fetuses inoculated with 1 ***×*** 10^6^ PFU of 68-1 RhCMV/FKBP-Rh108/SIVrtni survived until the scheduled tissue collection time point near term (Figure 2A). Amniotic fluid (AF) collected from all UCD59-inoculated pregnancies after fetal loss or when the surviving fetuses were collected by hysterotomy in the late third trimester contained very high levels of viral genome copies, particularly in fetuses that received the 1 ***×*** 10^6^ PFU dose (Figure 2B). Similarly, high genome copy numbers were also observed in the AF of the 2 dams with early fetal loss in the 68-1 RhCMV/SIVrtni-inoculated cohort, but only 1 of the remaining fetuses in this and the ΔRh110 cohort had detectable virus in the AF at the time of tissue collection. In contrast, RhCMV DNA was not detectable in the AF of 68-1 RhCMV/FKBP-Rh108/SIVrtni–inoculated fetuses. In surviving fetuses inoculated with UCD59, viral DNA was detected in all tissues, including the CNS, as indicated by genome copy numbers observed in each tissue sample (Figure 2C and Supplemental Figure 2) or by the average genome copy numbers measured per fetus (Figure 2D). Two of the 5 fetuses that survived inoculation with 68-1 RhCMV/SIVrtni did not have detectable viral DNA in most tissues, whereas the remaining 3 fetuses displayed high levels of viral genome copy numbers in multiple tissues, including the CNS (Figure 2C and Supplemental Figure 2). Viral genomes were discovered in non-CNS and CNS tissues of 9 fetuses inoculated with RhCMVΔRh110, including tissues recovered from 1 fetus found nonviable by ultrasound and 1 fetus recovered by early hysterotomy due to signs of impending demise (Figure 2C and Supplemental Figure 2). Together, these results are consistent with the fetal brain being particularly susceptible to viral replication both for PC-intact UCD59 and PC-deficient 68-1, even when deficient for pp71. In striking contrast, viral DNA was at or below the limits of detection in all non-CNS and CNS tissues of 68-1 RhCMV/FKBP-Rh108/SIVrtni–inoculated fetuses (Figure 2C and Supplemental Figure 2). Thus, Rh108-deficient RhCMV is unable to spread in vivo, even in immunologically immature fetal hosts, consistent with an inability to generate infectious progeny from initially infected cells, a vaccine strategy that would be safe for administration during pregnancy.

### Rh108-deficient RhCMV superinfects naturally RhCMV-infected RMs and elicits durable T_EM_.

**Figure 3:**
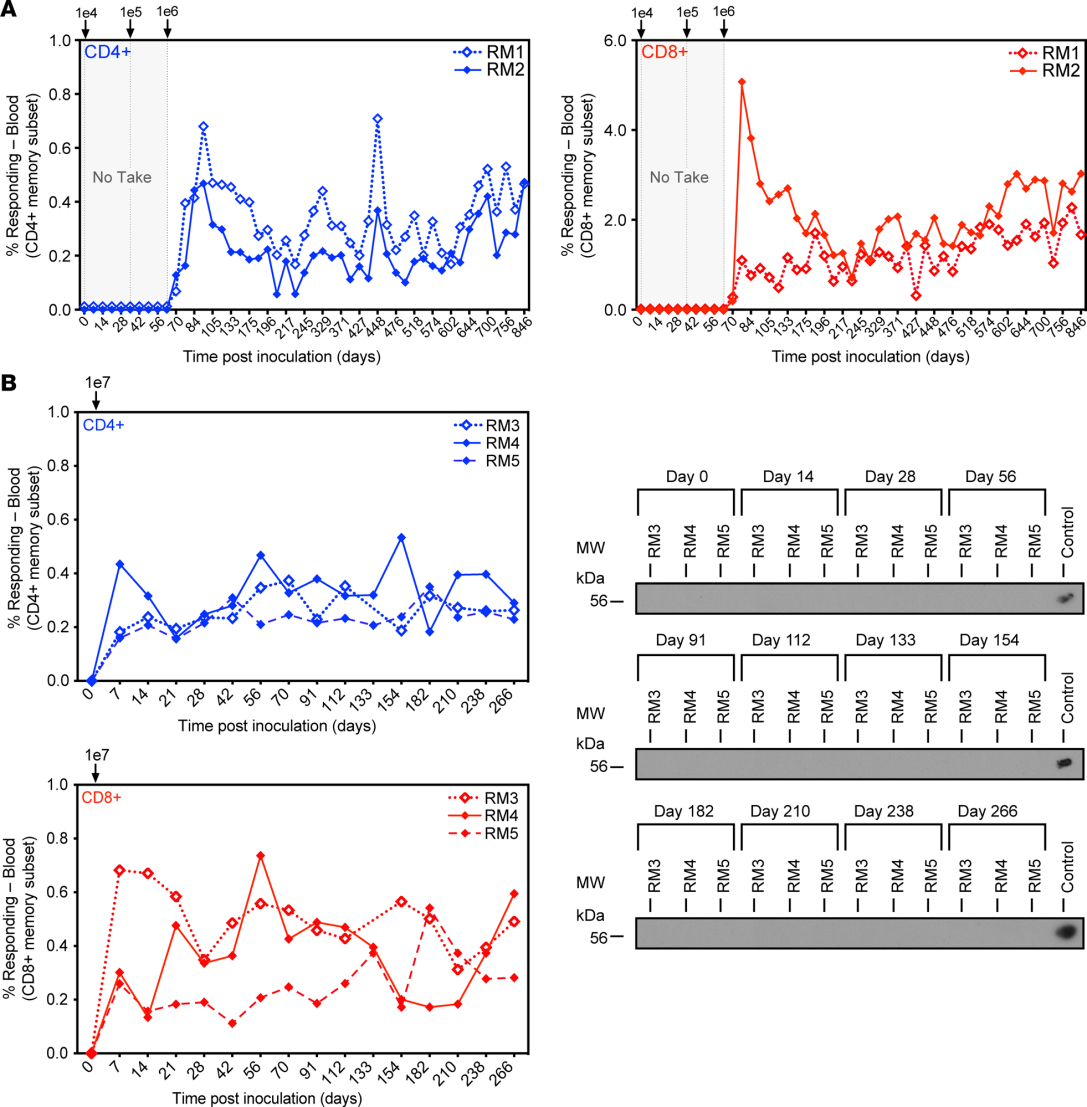
Rh108-deficient RhCMV/SIV induces SIV-specific T cell responses at high dose in the absence of vector shedding in urine. (**A**) Two RhCMV-seropositive RMs were sequentially inoculated with increasing doses of 68-1 RhCMV/FKBP-Rh108/SIVrtni until the onset of detectable SIV-specific CD4^+^ and CD8^+^ T cell responses in peripheral blood, as measured by intracellular cytokine staining (ICS) for IFN-γ and TNF-α using pools of 15 mer peptides overlapping by 11 amino acids (AA) corresponding to SIV Rev, Tat, and Nef (without Int) (10). The frequency of IFN-γ^+^and/or TNF-α^+^ memory T cells is shown for each of the indicated postinoculation time points. (**B**) Three additional RhCMV-seropositive RMs were inoculated with 1 ***×*** 10^7^ PFU of the same vector with longitudinal SIV Rev/Tat/Nef–specific T cell responses determined as described in **A** (left panels). Urine was collected from these 3 RMs at the indicated time points and, after concentration, cocultured with rhesus fibroblasts in the presence of *Shield-1* for 42 days. Cells were then lysed and analyzed by immunoblot for the presence of the SIV Rev/Tat/Nef/Int fusion protein using antibodies to the V5 epitope tag (Thermo Fisher Scientific) as described (23). The 68-1 RhCMV/SIVrtni lysates were used as positive controls. SIV Rev/Tat/Nef–specific T cell responses were also measured longitudinally in BAL fluid for RMs 1–5 (Supplemental Figure 3).

Single-cycle MCMV elicits MCMV-specific T_EM_ lasting for at least 1 year after inoculation and protects mice against reinfection with MCMV (28, 36, 37). Moreover, a single-cycle MCMV vector with FKBP fused to M79 and expressing an immunodominant epitope of the human papilloma virus E7 protein elicited E7-specific T cells and protected mice from tumor challenge at 13 months after immunization (41). Furthermore, conditionally replicating HCMV carrying N-terminal FKBP fusions on the essential transcription factor IE2 and the essential viral genome packaging factor UL51 (42) elicited robust T cell immunity to immunodominant HCMV proteins that lasted > 12 months after inoculating HCMV-naive human volunteers (43). However, all of these results were obtained in CMV-negative hosts. Since HCMV-based vectors need to be immunogenic regardless of HCMV-immune status, we determined whether and at what inoculation dose conditional single-cycle RhCMV vectors would elicit T cell responses in naturally RhCMV-infected RMs. We performed a dose-escalation study by inoculating 2 RhCMV^+^ RMs s.c. with sequential, ascending doses of 68-1 RhCMV/FKBP-Rh108/SIVrtni. We determined vector take by longitudinally monitoring SIV Rev/Tat/Nef–specific CD4^+^ and CD8^+^ T cell responses in peripheral blood and bronchoalveolar lavage (BAL) fluid using intracellular cytokine staining (ICS) for IFN-γ and TNF-α in response to overlapping SIV peptides (10, 14–17); responses to the SIV Int fragment were not monitored due to low immunogenicity (34). At 1 ***×*** 10^4^ and 1 ***×*** 10^5^ PFU, we did not observe SIV-specific T cell responses (Figure 3A and Supplemental Figure 3A), indicating that the vaccine was below the immunogenic threshold (15). However, robust SIV-specific CD4^+^ and CD8^+^ T cell responses were documented at doses of 1 ***×*** 10^6^ PFU (Figure 3A and Supplemental Figure 3A) or above (Figure 3B and Supplemental Figure 3B). Moreover, these responses were stable (or even slightly increasing) for more than 2 years of observation, the duration of the experiment, consistent with continuing antigen presentation (Figure 3A).

We demonstrated that deletion of *Rh110* encoding pp71, which counteracts host intrinsic immunity, resulted in a live-attenuated vector that was not shed in the urine of inoculated RMs (23). In contrast, WT RhCMV and 68-1 RhCMV–derived recombinants are continuously shed in urine (5, 10, 11, 16, 17, 23), although shedding is significantly reduced for 68-1 RhCMV (40). Similarly, we were unable to detect RhCMV/FKBP-Rh108/SIVrtni by immunoblot of *Shield-1*–treated fibroblasts cocultured with urine preparations from RMs 3–5 administered 1 ***×*** 10^7^ PFU (Figure 3B). The lack of shedding through 266 dpi suggests that the FKBP-degradation domain did not acquire mutations that restored viral spread and shedding in vivo; thus, degradation of Rh108 renders this vector permanently spread-deficient in vivo.

**Figure 4:**
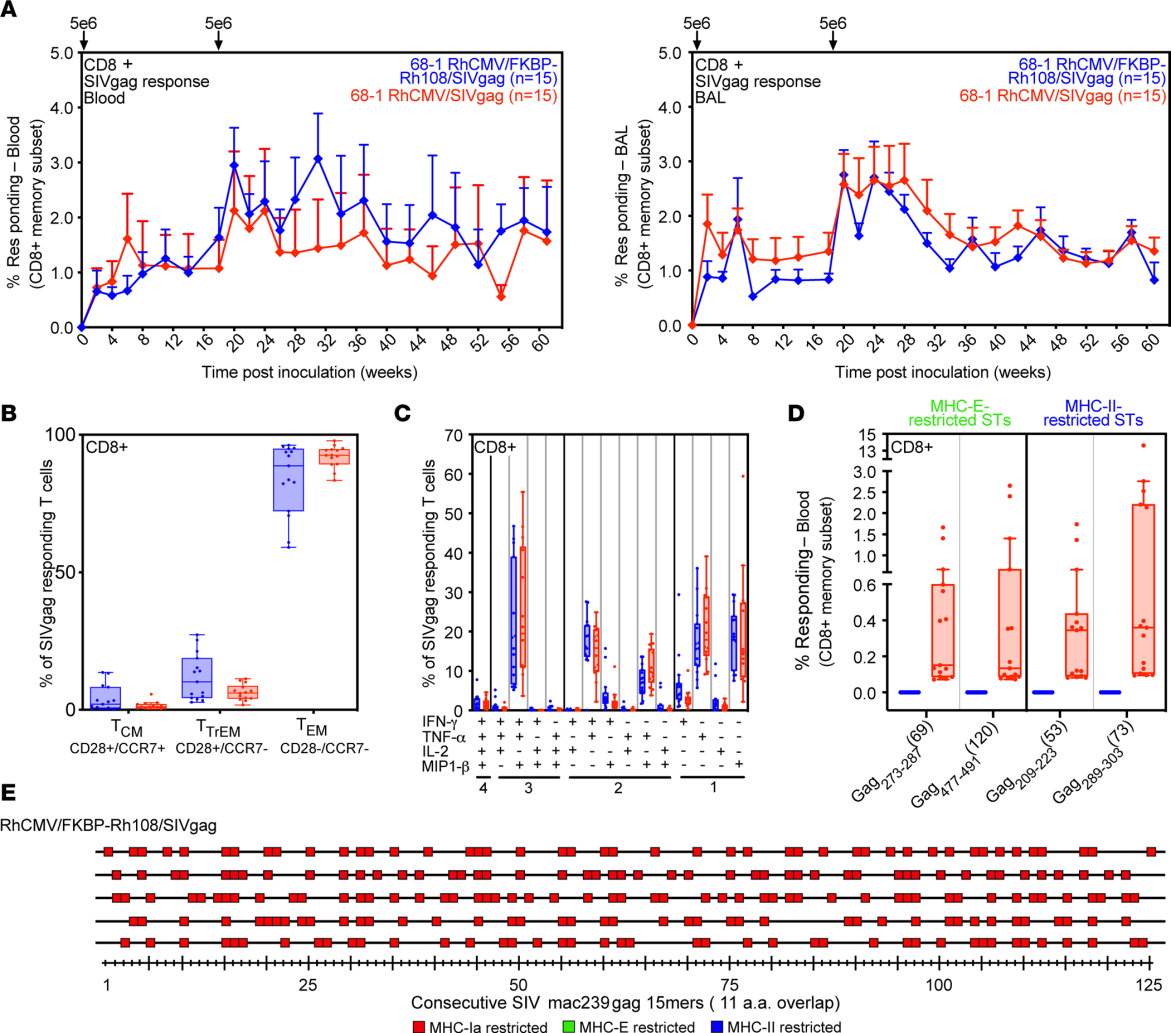
SIV-specific CD8^+^ T cells elicited by Rh108-deficient 68-1 RhCMV/SIVgag are similar in magnitude and functional phenotype to 68-1 RhCMV/SIVgag but differ in MHC restriction. (**A**) Mean (±SEM) CD8^+^ T cell frequencies as determined by ICS to overlapping (by 11 amino acids) SIV Gag 15mer peptides. Differences were NS between the 2 groups (Wilcoxon Rank Sum test of the AUC for each RM). (**B**) Box plots compare the memory differentiation phenotype of CD8^+^ T cells in blood during plateau phase (>60 weeks). Memory differentiation state (percentage of each subset within the total response) based on CD28 and CCR7 delineates central memory (TCM), transitional effector-memory (TTrEM), and effector-memory (TEM). Wilcoxon Rank Sum test was used to compare Gag-specific T cells within each subset (*P* > 0.05 for all comparisons). (**C**) Box plots compare blood CD8^+^ memory T cell frequencies responding to Gag 15mer peptides with TNF-α, IFN-γ, IL-2, and MIP-1β production, alone and in all combinations. Results were grouped according to the number of cytokines secreted. Wilcoxon Rank Sum test with multiple testing correction was used to compare Gag-specific T cells expressing 1, 2, 3, or 4 cytokines (*P* > 0.05 for all comparisons). (**D**) Plateau phase of blood CD8^+^ T cell responses to individual MHC-E– and MHC-II–restricted Gag supertopes (19). Box plots in **B**–**D** show jittered points and a box from first to third quartiles and a line at the median, with whiskers extending to the farthest data point within 1.5***×*** IQR above and below the box. (**E**) Plateau phase blood CD8^+^ T cells from RMs inoculated with 68-1 RhCMV/FKBP-Rh108/SIVgag were assessed by ICS for each of 125 consecutive Gag peptides. Responses > 0.05% (after background subtraction) are indicated by a box. Colors reflect MHC restriction based on response inhibition with MHC-E blocking peptide, MHC-II blocking mAb, and/or pan-MHC-I blocking mAb.

We next quantitatively and qualitatively compared the SIV-specific CD8^+^ T cell response elicited by a 68-1 RhCMV/FKBP-Rh108 to that elicited by the efficacious parental 68-1 vector, with both vector types expressing SIV Gag (*n* = 15 naturally RhCMV-infected RMs each). As shown in Figure 4A, there was no significant difference in the magnitude or durability of either the CD4^+^ or CD8^+^ Gag-specific T cell responses elicited by these 2 vectors over 60 weeks of observation. CD8^+^ T cell responses in RMs naturally infected with RhCMV or inoculated with RhCMV-based vectors are characterized by a highly EM-biased phenotype and a cytokine synthesis profile commensurate with this phenotype (TNF-α, IFN-γ, and MIP1-β, alone or combination, with little to no IL-2; refs. 10, 11, 14–17). Interestingly, despite the marked spread deficiency of the FKBP-Rh108/SIVgag vector, the plateau phase Gag-specific CD8^+^ T cell responses elicited by this vector were indistinguishable, with regard to EM bias and cytokine synthesis pattern, from responses elicited by 68-1 RhCMV/SIVgag (Figure 4, B and C). Therefore, single-cycle RhCMV elicits and maintains lasting T_EM_ similar to Rh108-intact RhCMV. This is a truly remarkable feature of a viral vectored vaccine that is unable to spread past the first inoculum–infected cells and suggests that a relatively small number of persistently infected cells (likely far less than the minimally immunogenic 1 ***×*** 10^6^ PFU dose) is sufficient to maintain these responses and drive their EM differentiation.

### Rh108-deficient RhCMV/SIV elicits MHC-Ia-restricted CD8^+^ T cells.

One of the most remarkable and surprising features of 68-1–based vectors is exclusive elicitation of CD8^+^ T cells recognizing peptides presented by MHC-II or the nonclassical MHC-Ib molecule MHC-E, instead of conventional MHC-Ia (18, 19). The ability of 68-1 RhCMV to elicit such unconventionally restricted T cells resulted from deletion or functional inactivation of 8 genes in 2 spatially distinct regions of the RhCMV genome analogous to the ULb’ region of HCMV that occurred spontaneously during prolonged tissue culture passage. These 8 genes include the above-mentioned PC subunits Rh157.5 and Rh157.4 (HCMV UL128 and UL130), as well as viral chemokine homologs of the HCMV UL146 family (16, 20). WT RhCMV, or 68-1 RhCMV in which these genes have been repaired, elicits MHC-Ia–restricted CD8^+^ T cells. However, 68-1 RhCMV lacking Rh110 maintained the ability to elicit unconventional CD8^+^ T cells (15, 23). Since FKBP-Rh108 RhCMV was based on 68-1, we determined whether this vector elicits unconventionally restricted CD8^+^ T cells.

**Figure 5:**
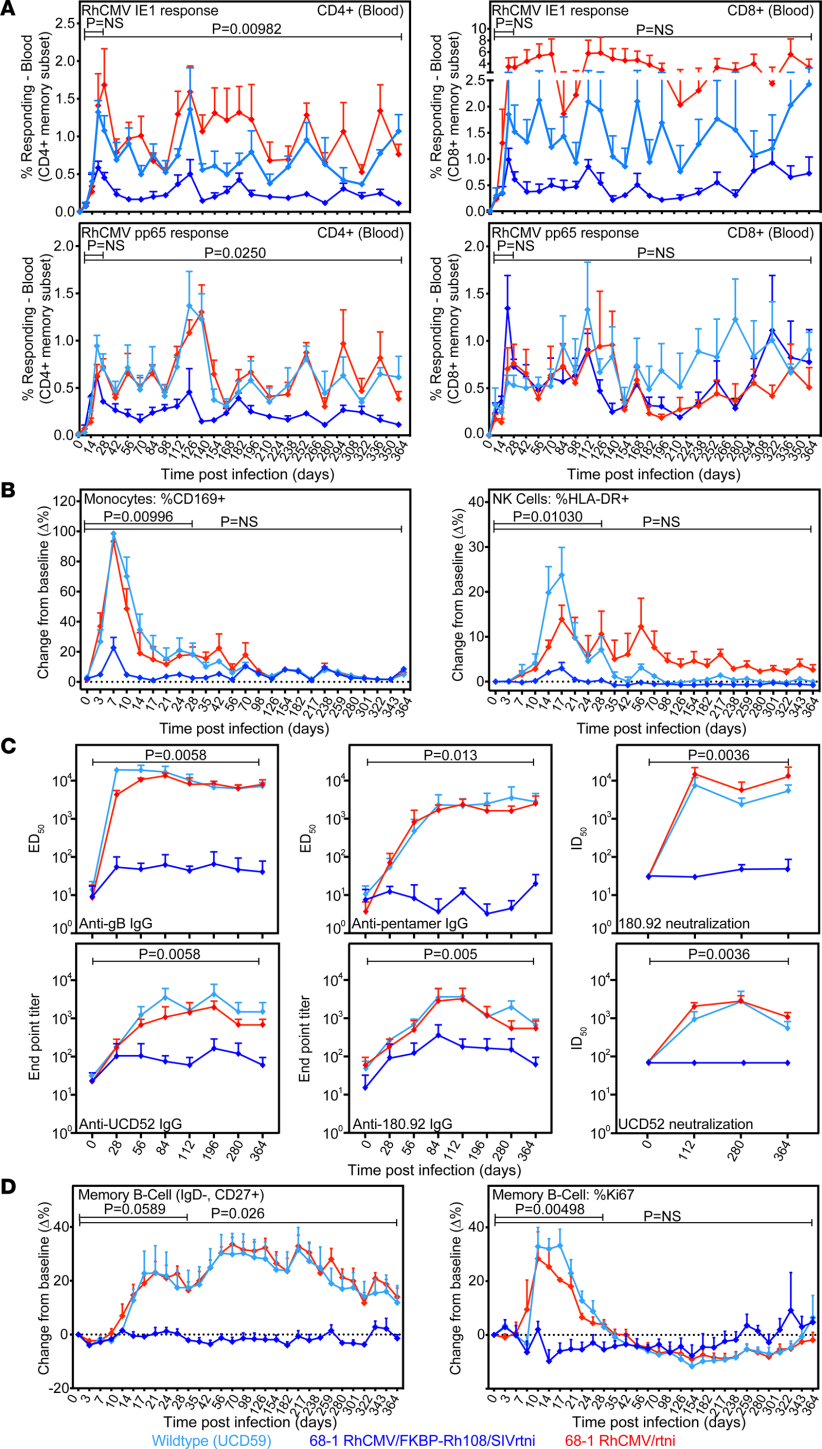
Limited innate immune and humoral responses to Rh108-deficient 68-1 RhCMV. RhCMV-seronegative RMs were inoculated in 3 groups of *n* = 4 each with 1 ***×*** 10^6^ PFU of indicated viruses. (**A**) ICS of CD4^+^ and CD8^+^ T cell response to consecutive overlapping RhCMV IE1 or pp65 15 mer peptide mixes in peripheral blood. The AUC over time for each animal was compared between the 3 groups by Kruskal-Wallis test with multiple testing correction via Bonferroni with a post hoc analysis assessing AUC over the first 28 days. (**B**) Monocyte (CD14^+^, HLA-DR^+^) and NK cell (CD8^+^, CD16^+^, CD3^–^) activation in blood by flow cytometric analysis of CD169 and HLA-DR, respectively. Results are shown as the mean difference (Δ%) ± SEM between the percentage of cells positive for each marker at the indicated time points compared with baseline. Statistical analysis was done as in **A**. (**C**) ELISA of IgG binding end point titers (mean ± SEM) to purified virions of UCD52 or 180.92, or to purified RhCMV glycoprotein B (gB) or pentameric complex (ED_50_; mean ± SEM). Neutralization titers (ID_50_; mean ± SEM) were determined for UCD52 on epithelial cells and for 180.92 on fibroblasts (65). Significance was determined by comparing the AUC after log_10_ transformation for each RM by Kruskal-Wallis test with FDR adjustment for multiple testing. Pairwise comparison between FKBP-Rh108 with the other 2 cohorts combined using the Wilcoxon Rank-Sum test with FDR adjustment resulted in *P* = 0.004. (**D**) B cell dynamics were determined by flow cytometric phenotyping, including the percentage of total memory B cells among small lymphocytes and within the memory B cell population, and the percentage of cells expressing the proliferation marker Ki-67. Results are shown as the mean difference (Δ%) ± SEM between the percentage of cells positive for each marker compared with baseline. Statistical significance was assessed as in **A**.

Some epitopes recognized by 68-1 RhCMV–elicited MHC-E– or MHC-II–restricted CD8^+^ T cells were shared among all vaccinated RMs (18, 19). Responses to such “supertopes” indicate unconventional CD8^+^ T cell epitope targeting. However, CD8^+^ T cells in peripheral blood mononuclear cells (PBMC) of fifteen 68-1 RhCMV/Rh108-FKBP/SIVgag–inoculated RMs (Figure 4D) or 5 RMs inoculated with 68-1 RhCMV/Rh108-FKBP/SIVrtni (Supplemental Figure 4) did not respond to either the MHC-E– or the MHC-II–restricted supertope peptides in SIV Gag or Nef, respectively. To determine whether lack of responses was supertope-specific or due to a general lack of unconventionally restricted CD8^+^ T cells, we deconvoluted the SIV Gag– or SIV Nef–specific CD8^+^ T cell response to the 15 mer peptide level in 5 RMs and then used MHC-specific blocking analysis to determine the MHC molecule presenting each of the 15 mer–specific responses, as described (16, 19). Strikingly, all 1 5mer peptide responses for either Gag (Figure 4E) or Nef (Supplemental Figure 4A) were blocked by the pan–MHC-I mAb, but not by the MHC-E–specific peptide VL9 or by MHC-II–specific mAb, indicating MHC-Ia restriction (16, 19). Thus, the Rh108-FKBP vector elicits only conventionally MHC-Ia–restricted CD8^+^ T cells, similar to pentamer-intact RhCMV or to RhCMV expressing the aforementioned viral chemokine-like genes.

Reduced L gene expression could limit the ability of virally infected cells to activate T cells due to reduced antigen expression or presentation. For instance, viral VL9 provided by the early protein Rh67 is required for MHC-E–dependent peptide presentation by supporting MHC-E egress (21). To explore the latter possibility, we monitored activation of MHC-E–restricted, RhCMV-specific CD8^+^ T cells to RhCMV-infected fibroblasts, thereby taking advantage of the fact that, unlike classically MHC-Ia–restricted T cells (5), MHC-E–restricted T cells recognize RhCMV-infected cells in vitro (21). As controls, we infected cells either with 68-1 RhCMV or with Rh67-deleted 68-1 RhCMV. As expected, T cells were stimulated by 68-1 RhCMV, and this was blocked by adding VL9 peptide, consistent with MHC-E restriction, whereas T cells were not simulated by Rh67-deficient RhCMV (21) (Supplemental Figure 5, A and B). Interestingly, MHC-E–restricted T cell stimulation by FKBP-Rh108 expressing RhCMV was dependent on *Shield-1*. Although fibroblasts were equally infected in the absence of *Shield-1*, T cell simulation was not observed (Supplemental Figure 5C). Unless T cell responses exclusively targeted late antigens expressed at lower levels without *Shield-1*, which is an unlikely scenario given that responses to IE and L antigens were present in all donor RMs, these observations suggest that the inability of Rh108-deficient RhCMV to elicit unconventionally restricted CD8^+^ T cells might be due to a lack of late genes required for antigen processing for MHC-E and, by extension, MHC-II.

### Rh108-deficient RhCMV infection shows reduced innate immune activation and anti-RhCMV antibody responses during primary infection.

To determine innate immune activation and de novo anti-RhCMV antibody (Ab) and T cell responses, we inoculated 4 RhCMV-naive RMs each with 1 ***×*** 10^6^ PFU of 68-1 RhCMV/FKBP-Rh108/SIVrtni, or for controls, 4 RMs with 1 ***×*** 10^6^ PFU of 68-1 RhCMV/SIVrtni, or 4 RMs with 1 ***×*** 10^6^ PFU of UCD59. We then monitored RhCMV pp65– and IE-specific T cells (Figure 5A), phenotypic evidence of monocyte and NK cell activation (Siglec-1/CD169 and HLA-DR induction; Figure 5B), and induction of RhCMV-specific Abs (Figure 5, C and D). The latter included measuring Abs binding to whole RhCMV virions — either the isolate UCD52 (40) or the fibroblast-adapted strain 180.92 (44) — to the purified RhCMV glycoprotein B (gB) or to the purified RhCMV PC consisting of gH, gL, Rh157.5, Rh157.4, and Rh157.6 (homologs of UL128, UL130 and UL131A). We also determined the ability of Abs to neutralize UCD52 or 180.92 RhCMV, as previously described (45, 46).

As expected, since the 1 ***×*** 10^6^ PFU dose of 68-1 RhCMV/FKBP-Rh108/SIVrtni elicited SIV-specific T cells in naturally RhCMV-infected RMs (Figure 3A), this dose induced pp65- and IE-specific T cell responses in RhCMV-seronegative RMs. The frequencies of CD4^+^ RhCMV–specific T cell responses were reduced relative to parental 68-1 and UCD59 infections but remained stable over time (Figure 5A). The reduced CD4^+^ T cell responses likely reflect a difference in antigen load between replicating and single-cycle vectors that is much more pronounced during primary infection compared with infection of CMV-immune animals. Peak monocyte and NK cell activation was significantly reduced in RMs administered 68-1 RhCMV/FKBP-Rh108 relative to the other RhCMVs as shown by post hoc statistical analysis of the first 28 days (Figure 5B). Moreover, whereas the parental 68-1 vector and UCD59 virus elicited robust RhCMV-specific binding and neutralizing Abs, 68-1 RhCMV/FKBP-Rh108 elicited limited anti-RhCMV binding IgG responses and no detectable neutralization responses (Figure 5C). It is interesting that 68-1 RhCMV was as efficient at eliciting Abs as UCD59, despite the fact that 68-1 viremia during primary infection is much reduced compared with UCD59 (40). This included Ab responses to the PC despite 2 of the subunits missing in 68-1, potentially indicating responses against the gH and gL components. Thus, the increased antigen load provided by UCD59 did not further increase Ab responses, suggesting that both UCD59 and 68-1 RhCMV provided saturating levels of antigen in RhCMV-naive animals at this dose. In contrast, the lack of RhCMV-specific Ab responses in 68-1 RhCMV/Rh108-FKBP/SIVrtni–inoculated RMs is consistent with low antigen load resulting from the inability of single-cycle RhCMV to spread in vivo, leading to failure of RhCMV-specific B cell response induction. This conclusion is supported by the observation that, in contrast to the robust initial B cell proliferation and memory B cell expansion in RMs given 68-1 RhCMV or UCD59, the 68-1 RhCMV/FKBP-Rh108/SIVrtni–vaccinated RMs showed no measurable B cell proliferation or induction of memory B cell expansion (Figure 5D).

### Rh108-deficient RhCMV/SIV does not protect against SIV challenge.

T cell responses elicited by 68-1 RhCMV/SIV are uniquely capable of controlling SIV infection upon repeated low-dose intrarectal or intravaginal challenge with the highly pathogenic SIV_mac239_ clone (10, 14, 16, 17). Moreover, this ability to arrest SIV replication in early primary infection was also observed for live-attenuated, pp71-deficient 68-1 RhCMV (15). In contrast, 68-1 RhCMV–derived vectors that have lost the ability to elicit MHC-E–restricted CD8^+^ T cells are no longer protected against challenge with highly virulent SIV_mac239_ (16, 21). The finding that this single-cycle RhCMV lacked the ability to elicit unconventional CD8^+^ T cells presented another opportunity to determine whether an attenuated vector that was genetically identical to 68-1 with respect to Rh67 and deletions of PC subunits and UL146-homologs, but that was unable to elicit MHC-E–restricted CD8^+^ T cells, would maintain the ability to protect against SIV challenge.

**Figure 6:**
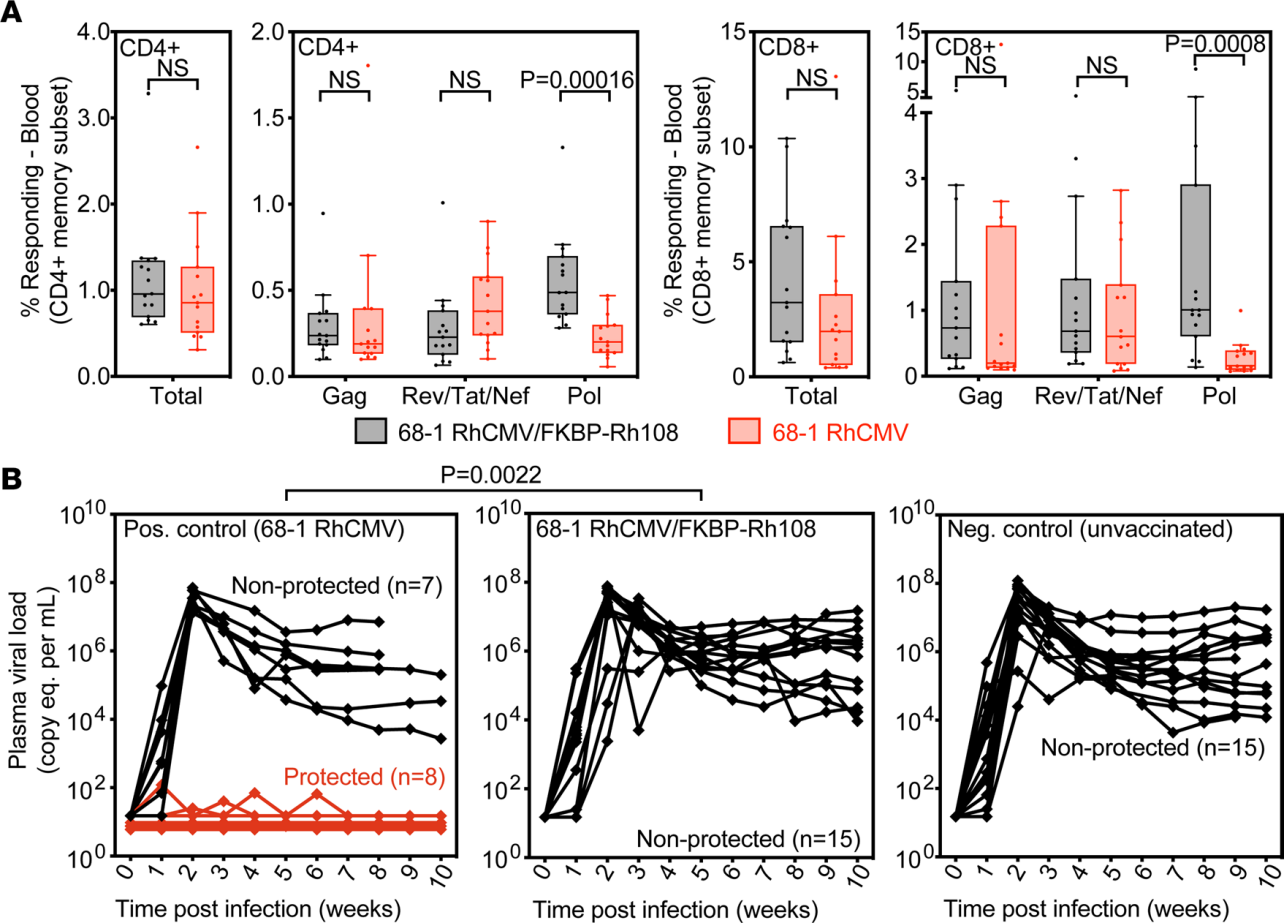
Rh108-deficient RhCMV/SIV vectors do not protect against SIV challenge. Fifteen RhCMV seropositive RMs were s.c. vaccinated at weeks 0 and 18 with a 3-vector set of 68-1 RhCMV/FKBP-Rh108 vectors individually expressing SIV Gag, Rev/Tat/Nef, and Pol (each vector given at 5 × 10^6^ PFU). After first vaccination, RMs were followed for 78 weeks prior to initiation of repeated, limiting dose SIV_mac239_ challenge. Previously published cohorts of 15 RMs vaccinated twice (weeks 0 and 18 with the 68-1 RhCMV/SIV 3-vector set; SIV Gag, Rev/Tat/Nef and 5′-Pol; 5 × 10^6^ PFU per vector) or left unvaccinated were used as positive and negative controls, respectively (16). (**A**) Comparison of the prechallenge SIV-specific CD4^+^ and CD8^+^ T cell response magnitude of the FKBP-Rh108 RhCMV/SIV vector–vaccinated RMs (week 78) to plateau phase responses of the Rh108-intact 68-1 RhCMV/SIV–vaccinated RMs (mean from week 61 to 80). Box plots show jittered points and a box from first to third quartiles and a line at the median, with whiskers extending to the farthest data point within 1.5***×*** interquartile range above and below the box. Statistical significance between the 2 groups was determined by Wilcoxon Rank-Sum test with multiple testing correction via FDR. (**B**) Outcome of SIV_mac239_ challenge after establishment of infection with repeated limited dose challenge. As previously described (16), SIV infection was documented in the protected RMs in the Rh108-intact 68-1 RhCMV vector vaccinated group by the de novo development of SIV Vif–specific T cell responses, detection of SIV infection in tissues by PCR, and adoptive transfer of leukocytes to naive RMs. Statistical significance between the 68-1 RhCMV/SIV cohort and the 68-1 RhCMV/FKBP-Rh108/SIV cohort was determined by Fisher’s exact test.

To address this question, 15 seropositive RMs were vaccinated with a Rh108-FKBP RhCMV vector set, composed of 3 vectors individually expressing SIV Gag, Rev/Tat/Nef, and polymerase (Pol). These 3 vectors were administered s.c. at 5 ***×*** 10^6^ PFU in 3 separate sites at weeks 0 and 18. RMs were then followed for 77 weeks prior to the initiation of repeated, limiting dose SIV_mac239_ challenge. A previously reported cohort of 15 RhCMV^+^ RMs vaccinated s.c. with a 3-vector set of 68-1 RhCMV expression SIV Gag, Rev/Tat/Nef, and 5′-Pol was used as a positive control (16). As shown in Figure 6A, the overall magnitude of the prechallenge SIV-specific T cell responses elicited by the Rh108-FKBP RhCMV vector set was as high or higher than the plateau phase responses of parental 68-1 RhCMV/SIV–vaccinated RMs. Of note, the increased frequency of SIV Pol–specific T cells in the RMs vaccinated with Rh108-FKBP RhCMV/SIV is at least in part due to incorporation of a longer Pol insert (912 amino acids [AA]) in Rh108-FKBP RhCMV compared with the 5′-Pol insert (349 AA) in the control 68-1 RhCMV vaccine.

Both vaccinated cohorts and an unvaccinated negative control cohort were subjected to the same repeated, limiting dose, intrarectal SIV_mac239_ challenge, with take of infection monitored by de novo induction of SIV Vif–specific T cell responses (Vif is not present in the vaccine inserts; thus, such responses are derived from the SIV infection) and plasma viral load (pvl) (10, 14, 16, 17). While the 68-1 vector resulted in replication arrest–type protection in 8 of 15 vaccinees (53%), no replication arrest was observed in the 15 RMs vaccinated with the Rh108-FKBP RhCMV vector set. Moreover, the early plateau phases of SIV pvl in these RMs were no different from those of the unvaccinated controls (Figure 6B). Thus, despite robust SIV-specific CD8^+^ T cell response frequencies, these MHC-Ia–restricted responses failed to mediate protection against SIV challenge.

## Discussion

We previously reported that RhCMV vectors lacking Rh110 — the homologue of HCMV UL82, encoding the tegument protein pp71 — elicited robust and long-lasting T cell responses in RhCMV^+^ RMs at doses at or above 1 ***×*** 10^4^ PFU (23). In contrast to the conditionally Rh108-deficient RhCMV studied here, RhCMVΔRh110 is still able to infect new target cells at high MOI and spread upon direct inoculation of fetal RMs. Thus, this vector is spread-impaired rather than spread-negative/single cycle. Given that 1 ***×*** 10^6^ PFU were required for Rh108-deficient RhCMV to elicit a measurable T cell response to the inserted SIV antigens, the level of attenuation of Rh108-deficient RhCMV appears to be ~100-fold more severe than that of the Rh110-deleted RhCMV. Thus, a 1 ***×*** 10^4^ PFU inoculation dose of the Rh110-deleted virus can spread sufficiently in vivo to increase infected cell burden to the same level of antigenic stimulation mediated by a 1 ***×*** 10^6^ dose of the nonspreading Rh108-deficient RhCMV. In both cases, these minimal immunogenic doses reflect a threshold above which the magnitude of the T cell responses was similar to those elicited by the spread-competent parental 68-1 RhCMV, which has a minimal immunogenic dose of ≤ 1 ***×*** 10^1^ PFU (5). However, while the inoculum dose is greatly amplified upon in vivo replication of spreading-competent vectors, this is not the case for single-cycle vectors.

The finding that single-cycle RhCMV elicits and maintains durable, high frequencies of T_EM_, despite being unable to spread from the initial infection sites, is consistent with previously reported lasting T cell responses elicited by single-cycle and highly spread-deficient MCMV in CMV-negative mice (28, 36, 41). Furthermore, recent reports from phase I clinical studies in seronegative volunteers using a conditionally replicating HCMV similarly described the elicitation of lasting T cell responses with effector phenotype (43). While the T cell phenotype elicited by conditionally replicating HCMV differed somewhat from that found in chronically infected individuals (43), the T cell phenotype observed in FKBP-Rh108 RhCMV–immunized RMs displayed a profile that is typical for RMs chronically infected with RhCMV with a strong EM bias (as defined by low CD28 and CCR7 expression) of CD8^+^ T cells and a mix of transitional and fully differentiated EM CD4^+^ T cells. The ability of CMV to elicit and maintain T_EM_ despite the complete absence of viral dissemination, and even in CMV-immune hosts, is a unique feature of CMV-based vaccines, suggesting that relatively few persistently infected cells suffice to continuously stimulate EM-biased T cell responses of exceptional frequency. This finding also suggests that efforts to use pharmacologic inhibition of HCMV replication to therapeutically reduce dominant CMV-specific EM responses in people (47) will likely fail due to the fact that extremely low antigen loads in the absence of viral replication and spread are still sufficient to maintain these responses.

Both innate immune activation and Ab responses to 1 ***×*** 10^6^ PFU doses of Rh108-deficient RhCMV were absent or much reduced from the 68-1 parent vector or WT RhCMV when given to RhCMV-naive RMs. In contrast, T cell responses were much less affected by vector attenuation. This finding is in agreement with the report that single-cycle MCMV elicited similar T cell but significantly reduced Ab responses compared with spreading-competent virus at a dose of 1 ***×*** 10^5^ PFU (36). The reduced humoral immunogenicity observed in these studies indicates that antigen levels sufficient for T cell immunogenicity are well below the threshold required for humoral immunogenicity. Since robust and durable Ab responses are elicited by conditionally replicating HCMV at doses of 3 × 10^7^ PFU (29), the poor humoral immunogenicity observed in our study can likely be overcome, at least in part, by increasing the dose of the vaccine inoculum. Indeed, increasing dose levels were reflected by increasing Ab response in human clinical trials with the HCMV vaccine, whereas T cell responses were not dose dependent (48). Since the main goal of our study was to elicit SIV-specific, protective T cell responses, we did not examine whether higher doses of the Rh108-deficient RhCMV would be able to elicit better Ab responses.

We demonstrated a lack of viral dissemination by direct high-dose inoculation of Rh108-deficient RhCMV into fetal RMs, a model that allows sensitive monitoring of RhCMV replication and dissemination in vivo in the absence of a mature immune system. This result was consistent with the lack of viral spread in tissue culture in the absence of *Shield-1* and suggests that an HCMV ortholog of this RhCMV vector (e.g., FKBP-UL79 HCMV) would make an extraordinarily safe vaccine — almost certainly unable to cause fetal disease or to reactivate in other settings of high-level immune deficiency. However, in the specific situation of an HIV-targeted vaccine, this safety came at the price of efficacy, as — despite an ability to generate high-magnitude and durable CD8^+^ T cell responses — Rh108-deficient RhCMV/SIV failed to protect against SIV. This efficacy failure resembles that observed in other RhCMV vector–based vaccines that, by virtue of expression of genetic inhibitors of unconventional response priming or tropism restriction, elicit MHC-I– and/or MHC-II–restricted CD8^+^ T cell responses. Indeed, lack of anti-SIV efficacy is a feature of all RhCMV vectors lacking MHC-E–restricted CD8^+^ T cell responses, with the data in this report further strengthening the association of MHC-E–restricted CD8^+^ T cell responses and SIV replication arrest–type protection (16, 17, 21).

We have previously shown that MHC-E– and/or MHC-II–restricted CD8^+^ T cell priming, mediated by direct antigen presentation by infected cells, dominates over presumably cross-primed conventional MHC-Ia restriction, when RhCMV vectors have the correct genotype for these unconventional responses (17). However, when MHC-E– and MHC-II–restricted CD8^+^ T cell priming is inhibited, MHC-Ia restriction becomes the default CD8^+^ T cell response type (16, 17). The FKBP-Rh108 RhCMV studied here has the correct 68-1 genotype for unconventional response priming, raising the question of how Rh108 deficiency prevents direct priming, resulting in an MHC-Ia–restricted CD8^+^ T cell response. One possibility is that the FKBP-Rh108 vector is unable to infect either the myeloid-derived cells required for MHC-E–restricted CD8^+^ T cell priming or the endothelial cells required for MHC-II–restricted priming, mimicking the MHC-Ia restriction observed for miR-126 and miR-142 dual tropism–restricted vectors precluded from productively infecting both cells types (17). However, there is no obvious mechanism for such cell type–selective inhibition of infection, and even if infection of these cell types was reduced by a nonspreading inoculum, the fact that increasing the FKBP-Rh108 dose by 10-fold failed to engender any unconventional priming (Figure 3B and Supplemental Figure 4) argues against this mechanism. Moreover, the finding that MHC-E–restricted responses can be elicited by single-cycle vectors with intact L gene expression, as discussed below, also argues against lack of myeloid cell infection being the underlying defect. The more likely possibility is that unconventional CD8^+^ T cell priming requires processes that occur during the late stage of the viral life cycle, which is unable to commence in the absence of Rh108. This possibility is supported by the finding that MHC-E–restricted T cell stimulation in vitro was dependent on Rh108 expression. Although the molecular underpinnings of this observation still need to be explored, we ruled out that this lack of stimulation was due to reduced expression of Rh67, an early transcript (Figure 1B). While a lack of late antigen expression could be responsible for the observed reduction of T cell stimulation in vitro, we consider this unlikely because both IE and L protein–specific T cells are present in 68-1 RhCMV–immunized RMs (Figure 5A). It is well documented that L gene products are required to reorganize subcellular vesicular structures for virion production and transport with substantial impact on vesicular trafficking to and from the cell surface (49, 50). Moreover, most viral antigens are expressed at the highest density in the late stage of the viral life cycle. Reorganization of intracellular membrane structures together with high antigen production might facilitate the exchange of MHC-E VL9 peptides and MHC-II CLIP peptides with viral peptides (including the SIV insert) in multivesicular endolysosomal vesicles with subsequent transport of the peptide-MHC complexes to the cell surface, potentially similar to MHC-II peptide loading in professional antigen-presenting cells (51). It is also possible that this presentation depends more specifically on 1 or more specific late genes that have yet to be elucidated. Further work will be required to distinguish between these various mechanistic possibilities.

While lack of MHC-E–restricted CD8^+^ T cell priming is almost certainly the primary deficit of the FKBP-Rh108 RhCMV/SIV vaccine, it might not be the only deficit, as efficacy also appears to depend on vaccine-induced innate immune responses that feature IL-15 signaling (52). It is possible that a single-cycle vector would be unable to elicit this necessary innate immune response. However, this does not seem to be the case, as preliminary analysis of complemented, gL-deleted RhCMV recombinants (53) indicates that single-cycle 68-1 RhCMV/SIV vectors blocked at the stage of new cell infection, but after a normal late stage, retained the ability to elicit unconventional CD8^+^ T cells and protect against SIV challenge (our unpublished observations). These data suggest that using Rh108/UL79 deficiency as an attenuation strategy for a CMV-based HIV/SIV vaccine is not viable due to the lack of MHC-E–restricted T cell responses. However, this does not mean that this approach would not be applicable to other vaccine applications for which MHC-E–restricted CD8^+^ T cell responses are not required. For example, protection against *Mycobacterium tuberculosis* (*Mtb*) was found to be independent of the CD8^+^ T cell program elicited by RhCMV vectors (13). While protection against *Mtb* or other infectious diseases by conditional single-cycle vectors still need to be demonstrated experimentally, studies in murine tumor models showed protection by MHC-Ia–restricted CD8^+^ T cells elicited by similarly attenuated MCMV-based vectors expressing tumor antigens (41, 54, 55). If experiments with FKBP-Rh108–attenuated RhCMV vectors confirm their ability to protect against infectious diseases such as *Mtb*, FKBP-UL79 fusions could be used as attenuation strategies for HCMV-based T cell vaccines targeting infectious diseases that are susceptible to T cell control — e.g., HPV, HBV or HCMV itself — as well as cancer, particularly in HCMV-seronegative, pregnant, or immunocompromised individuals.

## Methods

Supplemental Methods are available online with this article.

### Statistics.

Statistical information is available in Supplemental Methods.

### Study approval.

All protocols were approved prior to implementation by the respective Institutional Animal Care and Use Committees at the UCD and Oregon Health and Science University. Both institutions are Association for Assessment and Accreditation of Laboratory Animal Care accredited. Activities related to animal care (diet, housing) and screening animals prior to assignment to the study were performed as per standard operating procedures.

## Author contributions

KF and LJP designed and oversaw the study, coordinated collaborations, interpreted the results, and wrote the manuscript. SGH supervised the immunization experiments and performed T cell analysis with the help of CMH, ANS, RMG, KTR, and JCF. D. Malouli, PC, WP, EM, HT, NHVB, HCR, and JLW constructed, generated, and/or characterized recombinant RhCMV. RFI and D. Morrow performed in vitro T cell assays with RhCMV-infected cells. PAB and AFT designed and performed the fetal inoculation experiments and edited drafts of the manuscript, and KAS assisted with quantitative PCR (qPCR) anaysis of fetal and maternal samples collected. SRP and CEO analyzed the anti-RhCMV Ab responses. RB and CC performed statistical analysis.

## Supplementary Material

Supplemental data

## Version 1 02/07/2023

In-Press Preview

## Version 2 03/22/2023

Electronic publication
