# Supplementary material for: Late gene expression–deficient cytomegalovirus vectors elicit conventional T cells that do not protect against SIV
Source: JCI Insight. 2023 Mar 22;8(6):e164692. doi: 10.1172/jci.insight.164692 (PMC10070102; doi:10.1172/jci.insight.164692)
Supplement: Supplemental data [file jciinsight-8-164692-s152.pdf]

Supplemental Data File

Late gene expression-deficient cytomegalovirus vectors elicit conventional T cells that do not protect against SIV

Authors:

Scott G. Hansen[1], Jennie Womack[1], Wilma Perez[1], Kimberli A. Schmidt[2], Emily Marshall[1], Ravi F. Iyer[1], Hillary Cleveland Rubeor[1], Claire E. Otero[3,4], Husam Taher[1], Nathan H. VandeBurgt[1], Richard Barfield[5,6], Kurt T. Randall[1], David Morrow[1], Colette M. Hughes[1], Andrea Selseth[1], Roxanne M. Gilbride[1], Julia C. Ford[1], Patrizia Caposio[1], Alice F. Tarantal[2,7], Cliburn Chan[5,6], Daniel Malouli[1], Peter A. Barry[2], Sallie R. Permar[3,4], Louis J. Picker[1]*, Klaus Früh[1]*

[1]Vaccine and Gene Therapy Institute and Oregon National Primate Research Center, Oregon Health & Science University, Beaverton, OR, USA
[2]California National Primate Research Center, University of California, Davis, CA, USA
[3]Duke Human Vaccine Institute, Duke University Medical School, Durham, NC, USA
[4]Department of Pediatrics, Weill Cornell Medicine, New York, NY, USA
[5]Department of Biostatistics and Bioinformatics, Duke University Medical Center
[6]Center for Human Systems Immunology, School of Medicine, Duke University
[7]Departments of Pediatrics and Cell Biology and Human Anatomy, School of Medicine, University of California, Davis, CA, USA

*co-corresponding authors: Klaus Früh, Tel.: 503 418 2735, email: fruehk@ohus.edu; Louis Picker, Tel.: 503 418 2720, email: pickerl@ohsu.edu. Address: 505 NW 185th Ave, Beaverton, OR, 97006, USA

Supplemental Methods

RhCMV strains and recombinants. All RhCMV recombinants were derived from BAC-cloned 68-1 RhCMV (1, 2) (Genbank MT157325). 68-1 RhCMVΔRh110/SIVrtni was described previously (3). Recombinants were propagated on primary rhesus fibroblasts (RF) or telomerized rhesus fibroblasts (TRF) (4). Complemented 68-1 RhCMVΔRh110/SIVrtni was grown on TRF stably expressing RhCMV pp71 (3). UCD59 and UCD52 are low passage primary isolates propagated on monkey kidney epithelial cells to preserve the PC and epithelial cell tropism (5). The sequences of UCD59 and UCD52 were recently determined (6) (respective Genbank numbers: MT15733 and MT157330.1). The 180.92 isolate is a mixture of the previously reported genome sequence that contains deletions in the genomic region homologous to the HCMV UL-b' region (7) (Genbank # DQ120516) and a minority population with an intact genome (8). This strain was propagated on TRF. Virus stocks were generated and titered as described previously (6, 7, 9).

Recombinant 68-1 RhCMV expressing a V5-epitope tagged fusion protein of SIV_{mac239} Rev, Tat, Nef and Integrase (SIVrtni) was described (10, 11). Using a published recombination strategy (12) we fused the FKBP degradation domain (13) to the N-terminus of Rh108 by BAC mutagenesis to generate 68-1 RhCMV/FKBP-Rh108/SIVrtni. Briefly, a cassette containing the FKBP and Kanamycin resistance (KanR) genes flanked by FRT sites (kindly provided by Dong Yu, Washington University, St. Louis, MO) was amplified by PCR using 50 bp homology primers and inserted into the 5' end of the Rh108 ORF by linear recombination in the bacterial strain SW105 containing an arabinose-inducible Flp recombinase (14). Upon selection, the KanR cassette was removed by FRT recombination resulting in an in-frame FRT site between N-terminal FKBP and the Rh108 coding region.

To generate Rh108-FKBP expressing 68-1 RhCMV/SIV vectors we first inserted the FKBP degradation domain into the 5' end of Rh108 as above. Next, we inserted SIV_{mac239} Gag, or Rev/Tat/Nef fusion (15), or Pol (3) by linear recombination of a PCR fragment containing 50 bp homology arms corresponding to the 5' and 3' end of Rh19 and a cassette containing the respective SIV gene together with the KanR selectable marker flanked by the FRT-5 site (3). The Pol fragment spans the N-terminal 912 AA of the SIV polymerase ORF and contains mutations to inactivate protease (Δ25-DTG-27), reverse transcriptase (Δ184-YMDD-187), RNaseH (ΔE478), and integrase (ΔD64, ΔD116, and ΔE152) as described (16). The KanR cassette was removed by Flp-mediated recombination of the FRT-5 sites resulting in a residual, untranslated FRT-5 site. The final BACs were analyzed by restriction digest, Sanger sequencing of inserted regions and next generation sequencing of the entire genome on an Illumina MiSeq sequencer.

Recombinants were reconstituted by electroporation of BAC DNA into RF in the presence of 1 µM *Shield-1* (Takara). The loxP-flanked BAC cassette encodes Cre recombinase under control of the SV40 promoter resulting in spontaneous excision of the BAC cassette (17). Viral stocks for in vivo and in vitro experiments were generated and titers were determined by 50% tissue culture infective dose (TCID50) on TRF in the presence of 1 µM *Shield-1*. Expression of SIV antigens was confirmed by reverse transcription PCR (RT-PCR) (**Fig. S1B**).

Virologic Assays

Growth Curves: To measure *Shield-1*-dependent growth of RhCMV in single and multi-step growth curves, RF were infected in duplicates at a MOI of 3 or 0.01, respectively, in the presence or absence of 1 µM *Shield-1* treatment every third day. Viral titers in the supernatants were determined in triplicates by immunofluorescence assay (IFA) as described for HCMV (18). Briefly, RF were infected with a serial dilution of each harvested sample and, at 72h post infection, cells were fixed with 100% methanol at ≤-20°C and then stained with the anti-RhCMV pp65 antibody (19) for 1h at 37°C. Subsequently, bound antibodies were visualized with Alexa Fluor 488-conjugated goat anti-mouse IgG secondary Ab (Invitrogen). After 1h at 37°C, cells were washed three times with PBS before staining with DAPI for 20min at room temperature. Images were acquired using an EVOS fluorescence microscope (Life Technologies) and ImageJ software was used to process the images. The ratio of infected versus uninfected cells was used to calculate the focus forming units per milliliter (FFU/ml) for each corresponding sample.

Expression of RhCMV mRNA: RhCMV gene expression was determined by quantitative RT-PCR (20) upon infection of RF at an MOI of 3 in the presence or absence of *Shield-1*. The cells were harvested at 0, 4, 8, 12, 24, 36, and 48h post infection, total RNA was isolated using TRIzol (Ambion) and transcribed into cDNA using the Maxima Reverse Transcriptase (ThermoFischer Scientific) and oligo-dT 18 primer (Integrated DNA Technologies). qPCR reactions were performed using a StepOnePlus Real-Time PCR System (Applied Biosystems) and data were collected using StepOne Software v2.3. The following forward (F) and reverse (R) primers were used to generate a PCR fragment specific for each gene, which was then cloned into the pGEM-T Easy Vector (Promega): GAPDH (F:5‘-TTCAACAGCGACACCCACTCT-3’; R:5’-GTGGTCGTTGAGGGCAATG-3’), Rh38.1 (F:5’-CGGGACCCAGGGAAATG-3’; R:5’-TCGTCACCCTCAAGCGTTATG-3’), Rh67 (F: 5’-AGCAGTGTGCGGCATGAA-3’; 5’-GCCCCCCTTGCATGTAGAC-3’), Rh110 (F:5’-CACCTGTTACGCCGTATTCTTTC-3’; R:5’-GACCCGCGTCCATGCTAAT-3’), Rh137 (F:5’-GGCGCAACATACTACCCAGAA-3’; R:5’-GTAGCCATCCCCATCTTCCA-3’), Rh156 (F:5’-AGTATGCCAAGCCTCATATTAAGGA-3’; R:5’-GCATATGGTGCTTGCTCTTAGAAG-3’), Rh189 (F:5’-GGAGCGCCCGGTAAGG-3’; R:5’-CGATGGAGTTTTATGCTTTGCA-3’). Primer set-specific standard curves were generated from these plasmids for each indicated gene to calculate mRNA copy numbers. All transcript copy numbers were normalized to GAPDH. Results for each gene and time point were expressed as relative mRNA copy numbers.

Determining RhCMV genome copies in fetal tissue: Viral DNA genome copy numbers in AF and fetal tissues were determined by qPCR as described previously (21) to detect a 71 bp amplicon of the RhCMV gB/Rh89 ORF (F: 5’-TGCGTACTATGGAAGAGACAA-3’; R: 5’-ACATCTGGCCGTTCAAAAAAA-3’; Probe: 5’-FAM-TCCAGCCTCCATAGCCGGGAAGG-TAMRA-3’). Each sample was run in triplicate and a standard curve was generated by using 10-fold serial dilutions of a plasmid (10^6 to 10^0 copies per reaction) containing the gB amplicon. A sample was considered RhCMV-positive when at least 2 of the 3 wells had detectable signal.

Shedding of RhCMV: Shedding in urine was monitored by virus co-culture as described previously (3).

SIV-quantification: Quantitative assessment of SIV DNA and RNA in cells and tissues was performed using SIV_{mac239} Gag-targeted, nested quantitative hybrid real-time/digital RT-PCR and PCR assays, as previously described (22-24).

Antibody Measurements and Virus Neutralization Assays. RhCMV-specific IgG Ab kinetics were measured in plasma by ELISA using either whole virion preparations of RhCMV strains UCD52 and 180.92, or purified glycoprotein preparations of RhCMV gB or PC as described previously (25). The lower threshold for Ab reactivity was considered to be three standard deviations above the average OD measured in pre-infection, RhCMV-seronegative samples at the starting plasma dilution (1:30). The endpoint titer reported is the highest dilution of plasma that exceeds this positivity threshold. ED50 was calculated as the sample dilution where 50% binding occurs by interpolation of the sigmoidal binding curve.

Neutralization of RhCMV UCD52 or 180.92 was determined as described (25). The ID50 was calculated as the sample dilution that caused a 50% reduction in the number of infected cells compared with wells treated with virus only.

Rhesus Macaque studies. Direct IP inoculation of fetal RMs was performed in utero under ultrasound guidance as described previously (26, 27) at the California National Primate Research Center (CNPRC). Immunogenicity studies of single cycle RhCMV/SIV vectors and challenge studies with SIV_{mac239} were conducted at the Oregon National Primate Research Center (ONPRC). All RMs used were *Macaca mulatta* of Indian genetic background purpose bred at the CNPRC or ONPRC.

Fetal inoculation: Forty RhCMV-seropositive female RMs were time mated and identified as pregnant sonographically (28). They were selected for fetal inoculation during the late first trimester/early second trimester after confirming normal embryonic growth and development by ultrasound. Maternal health was monitored daily and all pregnancies were assessed by ultrasound across gestation to assess fetal growth and development, and monitored for the typical sequelae and teratogenic findings associated with RhCMV (26, 27). Dams were weighed each time they were sedated for ultrasound examinations. The dams were administered Ketamine Hydrochloride (10 mg/kg) for routine ultrasound examinations and Telazol (5-8 mg/kg) on the day of fetal IP inoculation. Post-inoculation, sonographic measurements of the fetal head, abdomen, and limbs, in addition to gross anatomical evaluations, were assessed weekly and all measures were compared to normative growth curves for rhesus fetuses (28). Fetal tissues were harvested at hysterotomy near term according to established methods (27). AF samples were collected after fetal nonviability was detected or at the time of tissue collection post-hysterotomy. Fetal body weights and body measures were assessed, and tissues were grossly evaluated. Select fetal tissues and the placenta were weighed. Sections of the primary and secondary disks were collected as well as umbilical cord, membranes, and decidua. Samples of all tissues were snap-frozen over liquid nitrogen. Representative sections of all tissues were also preserved in formalin, embedded and sectioned at 5-6 µm, and then stained with hematoxylin and eosin. Specimens from animals of comparable age without any interventions (controls) were similarly processed and analyzed in parallel. All dams were returned

to the breeding colony post-hysterotomy. All activities related to animal care were performed according to CNPRC standard operating procedures.

Immunogenicity and SIV challenge studies: 49 male and female RMs were used, including 19 RMs for immunogenicity analysis of single cycle RhCMV vectors or control RhCMV and 30 RM for efficacy analysis of single cycle or control RhCMV/SIV vaccines. RhCMV vectors were dosed SC at 10^4-10^7 PFU for immunogenicity analysis and $5x10^6$ PFU per vector for efficacy analysis. At assignment, RMs were free of Cercopithicine herpesvirus 1, D-type simian retrovirus, simian T-lymphotrophic virus type 1, and *Mycobacterium tuberculosis*. Twelve RMs used for immunogenicity studies were also negative for RhCMV whereas all others were naturally RhCMV-infected. RMs were housed in Animal Biosafety level (ABSL)-2 for immunogenicity studies and ABSL-2+ rooms for the challenge phase. Study RMs were both single and pair cage housed. RMs were only paired with one another during immunogenicity studies if they were from the same immunization group. All RMs were single cage-housed during the challenge phase due to the infectious nature of the study. SIVmac239 challenge virus was kindly provided by Brandon Keele (Frederick National Laboratory, Frederick, MD) Regardless of their pairing, all animals had visual, auditory and olfactory contact with other animals. Single cage-housed RMs received an enhanced enrichment plan that was designed and overseen by non-human primate behavior specialists. RMs were fed commercially prepared primate chow twice daily and received supplemental fresh fruit or vegetables daily. Fresh, potable water was provided via automatic water systems. Physical examinations including body weight and complete blood counts were performed at all protocol time points. RMs were sedated with ketamine HCl or Telazol for procedures, including intradermal and subcutaneous vaccine administration, venipuncture, BAL, bone marrow and lymph node biopsy, and SIV challenge. At humane or scheduled endpoints, RMs were euthanized with sodium pentobarbital overdose (>50 mg/kg) and exsanguinated via the distal aorta, and tissue collection at necropsy was performed by a certified veterinary pathologist.

T cell assays.

MHC-E-restricted $CD8^+$ T cell responses to RhCMV-infected cells were determined by flow cytometric ICS as described (29). Briefly, RF were infected at an MOI of 3 for 48h. Cells were harvested by trypsinization, resuspended at 4×10^6 cells/ml in DMEM plus 10% FBS. MHC-blocking reagents were added 2h before 50µl aliquots of these infected cell suspensions (2×10^5 cells) were added to 5×10^5 $CD8\beta^+$ T cells in 50µl. The T cells were isolated from pre-existing PBMC samples of 68-1 RhCMV-immunized RM using a non-human primate $CD8^+$ T cell isolation kit (Miltenyi Biotec) and LS columns (Miltenyi Biotec). ICS was performed as described below.

SIV-specific $CD4^+$ and $CD8^+$ T cell responses were measured in PBMC or BAL by flow cytometric ICS as described (16, 20, 23, 30, 31). T cell responses to total SIV antigens were measured using mixes of sequential 15-mer peptides (11 amino acid overlap) spanning the SIV_{mac239} Gag, Pol, Nef, Rev, Tat, and Vif proteins. T cell responses to individual peptides were measured similarly by ICS and the MHC restriction type (MHC-Ia, MHC-E, MHC-II) of a peptide response was determined by pre-incubating

isolated mononuclear cell aliquots with either the pan anti-MHC-I Ab W6/32 (10μg/ml) (Biolegend), the MHC-II-blocking Ab G46.6 (10μg/ml) (BD Bioscience), or the MHC-E blocking peptide VMAPRTLLL (VL9; 20μM). To be considered MHC-E-restricted, the individual peptide response must have been blocked by both W6/32 and VL9, and not blocked by anti-MHC-II. MHC-II-restricted responses were blocked by anti-MHC-II but not anti-MHC-I or VL9, and MHC-Ia-restricted responses were blocked by anti-MHC-Ia only (30, 31). Responses that did not meet these inhibition criteria were considered indeterminate.

Stained samples were analyzed on an LSR-II or FACSymphony A5 flow cytometer (BD Biosciences). Data analysis was performed using FlowJo software (Tree Star). In all analyses, gating on the lymphocyte population was followed by the separation of the $CD3^+$ T cell subset and progressive gating on $CD4^+$ and $CD8^+$ T cell subsets (**Fig. S6**). Antigen-responding cells in both $CD4^+$ and $CD8^+$ T cell populations were determined by their intracellular expression of CD69 and either or both of the cytokines IFN-γ and TNF-α (or in polycytokine analyses, expression of CD69 and any combination of the cytokines: IFN-γ, TNF-α, IL-2, MIP-1β). Assay limit of detection was determined as previously described (32), with 0.05% after background subtraction being the minimum threshold used in this study. After background subtraction, the raw response frequencies above the assay limit of detection were "memory-corrected" (e.g., % responding out of the memory population), as described (16, 20, 23, 30, 31). For memory phenotype analysis of SIV-specific T cells, all $CD4^+$ or $CD8^+$ T cells expressing CD69 plus IFN-γ and/or TNF-α were first Boolean OR gated, and then this overall Ag-responding population was subdivided into the memory subsets of interest on the basis of surface phenotype (CCR7 vs. CD28). Similarly, for polycytokine analysis of SIV-specific T cells, all $CD4^+$ or $CD8^+$ T cells expressing CD69 plus cytokines were Boolean OR gated and polyfunctionality was delineated with any combination of the four cytokines tested (IFN-γ, TNF-α, IL-2, MIP-1β) using the Boolean AND function.

Immunophenotyping. Whole blood lymphocytes were stained and analyzed using the software described above. Monocytes were selected based on light scatter properties then selected by $CD3^-$ $CD20^-$ $CD8^-$ HLA-DR^+ lymphocytes and divided into monocyte populations based on CD14 and CD16 expression. Upregulation of the activation marker CD169 was also measured on the Boolean OR of $CD14^+$ $CD16^-$ (classical), $CD14^+$ $CD16^+$ (intermediate), and CD14low $CD16^+$ (non-classical) combined populations (Fig S5A). Memory B cell subsets were defined by their small lymphocyte light scatter properties then selected by $CD3^-$ $CD20^+$ $CD27^+$ IgD- (memory) with additional analysis of proliferation by Ki67. NK cells were also defined by their small lymphocyte light scatter $CD3^-$ $CD20^-$ $CD14^-$ $CD8^+$ $NKG2A^+$ $CD16^+$ with activation followed by HLA-DR expression. The gating procedure used to identify these populations are shown in Fig S5B.

Statistical Analysis. For analyses involving more than two groups we first tested for any overall difference by Kruskal-Wallis test with multiple-testing correction via the Benjamini Hochberg false discovery rate (FDR) adjustment (33). When only two tests were being adjusted for, the adjusted p-value was set to minimum (p-value/2), the Bonferroni adjustment. Kruskal-Wallis p-values were derived via 100,000 permutations using the R package coin (34). Time to spontaneous abortion was compared via a

log-rank tests with post-hoc pairwise comparisons done as linear rank tests in the R coin package. Genome copy numbers for CNS tissues that were sampled multiple times in some experiments (**Fig. S2**) were averaged prior to comparing the mean genome copy numbers of CNS and non-CNS tissues. We did not include prostate, uterus or seminal vesicles in the calculation of the mean in non-CNS tissues. T cell assay results, immune cell phenotyping results and Ab assay results were first analyzed by Kruskal-Wallis test followed by pairwise comparisons using the Wilcoxon Rank Sum test with adjustments for multiple testing via Holm (35, 36). The T cell responses and phenotypes between the vaccinated SIV challenge cohorts were compared by Wilcoxon Rank Sum test with Benjamini Hochberg correction. The results of the SIV challenge experiments were analyzed by Fisher's exact test.

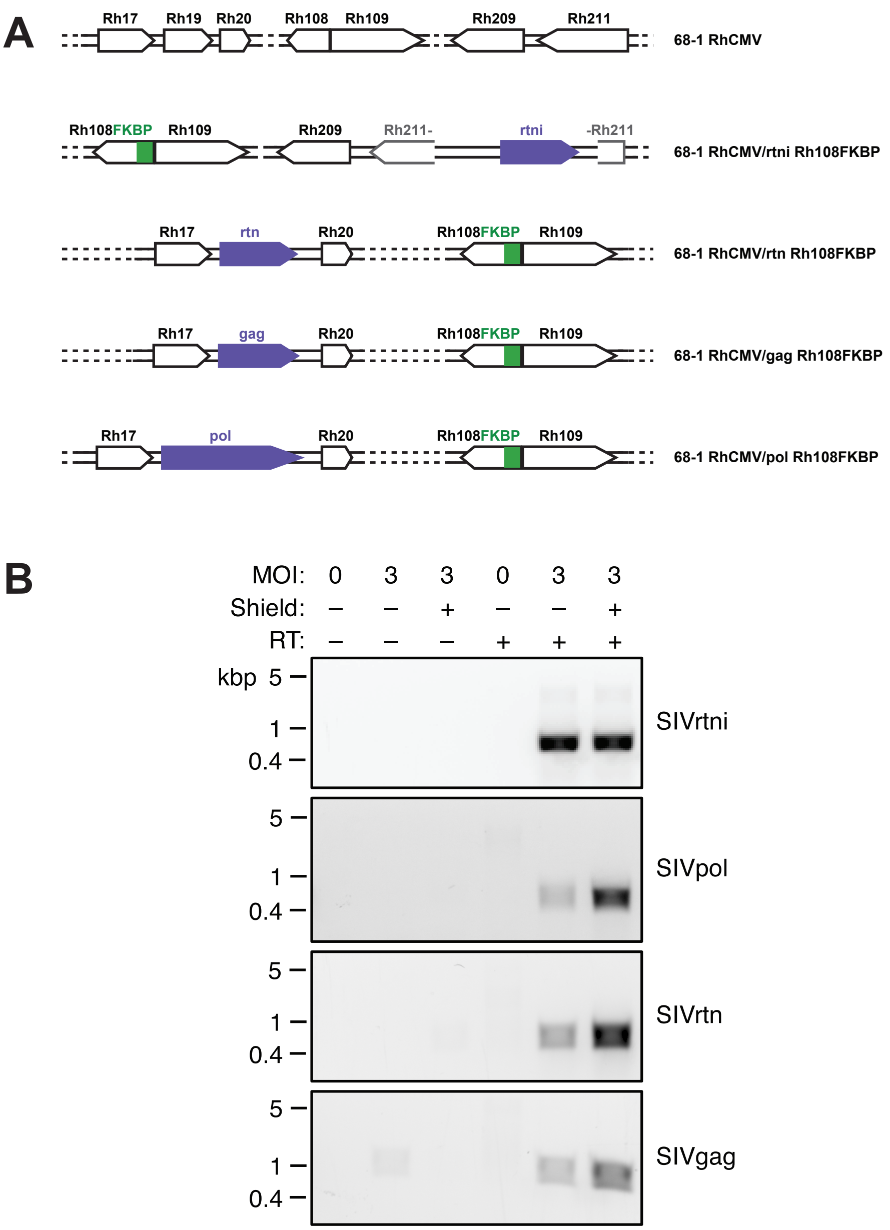

<u>Supplemental Figure 1</u>: Schematic diagram and SIV antigen expression by recombinant 68-1 RhCMV Rh108-FKBP constructs. (**A**) The FKBP-degradation domain was fused in-frame to the Rh108 amino-terminus of 68-1 RhCMV. SIV antigens were either expressed by inserting an expression cassette containing the cellular EF1α promoter into the gene Rh211 or by replacing the gene Rh19 thus using the endogenous promoter for expression. (**B**) Expression of SIV antigens was demonstrated by RT-PCR of rhesus fibroblasts at 48 hours post-infection with the indicated MOI in the presence or absence of 1 µM *Shield-1* using the following PCR primers specific for each insert: SIVrtni (493bp) F: 5'-CCGCATCCT-CGACATCTACC, R: 5'-GATTTCCTCCCCCAGGTTGG; SIVrtn (409bp) F:5'-AGCCATGAACGCGAAGAAGA, R:5':GTTTCCACAGCCAGCCAAAG; SIVgag (448 bp) F:5'-ATCTGGTGCATTCACGCAGA, R:5' ATCCTGACGGCT-CCCTAAGT; SIVpol(476bp) F:5'-GAGAATCACCGTGCTGGACA, R:5'-CCCAATTGAGGACTCCCACC.

Virus	UCD59 RhCMV					68-1 RhCMV/SIVrtni					68-1 RhCMV/Rh108-FKBP/SIVrtni								
Inocula Titer *(10⁶pfu)*	0.16	0.16	0.1	0.1	1.0	1.0	1.0	1.0	1.0	1.0	1.0	1.0	1.0	1.0	1.0	1.0	1.0	1.0	1.0
**Fetal Tissue**	RM1.1	RM1.2	RM1.3	RM1.4	RM2.1	RM3.1	RM3.2	RM3.4	RM3.5	RM3.6	RM4.1	RM4.2	RM4.3	RM4.4	RM4.5	RM4.6	RM4.7	RM4.8	RM4.9
**Sex**	M	F	M	F	F	F	F	M	M	M	F	F	F	M	F	F	F	M	F
Thymus	9	2	8	0	11	0	0	0	0	0	0	0	0	0	0	0	0	0	0
Spleen	6,092	151	202	1,141	540	0	0	0	0	0	0	0	0	0	0	0	0	0	0
Liver	17	11	66	1	45	0	0	0	0	0	0	0	0	0	0	0	0	0	0
Pancreas	1	2,025	467	253	1,322	0	38	0	0	0	0	0	0	0	0	0	0	0	0
Skin	15	120	79	6	921	0	1,090	0	0	0	0	0	0	0	0	0	0	0	0
Gonad-R	0	0	7	45	25	0		76,763	0	0	0	0	0	0	0	0	0	0	0
Gonad-L	0	0	179	2	34	0		80,764	0	0	0	0	0	0	0	0	0	0	0
Ax LN-R	710	9	258	21	286	0	69	0	0	0	0	0	0	0	0	0	0	0	0
Ax LN-L	0	28	237	9	537	0	41	0	0	0	0	0	0	0	0	0	0	0	0
Ing LN-R	48	47	51	100	724	0	1,483	0	0	0	0	0	1	0	0	0	0	0	0
Ing LN-L	217	12	82	47	396	0	0	68	0	0	0	0	0	0	0	0	0	0	0
Adrenal-R	16	0	0	0	7	0	0		0	0	0	0	0	0	0	0	0	0	0
Adrenal-L	0	0	0	26	34	0	0	0	0	0	0	0	0	0	0	0	0	0	0
Kidney-R	2,272	1,369	1,362	225	2,054	0	0	0	0	0	0	0	0	0	0	0	0	0	0
Kidney-L	336	2,745	2,149	254	1,300	0	0	0	0	0	0	0	0	0	0	0	0	0	0
Uterus		0		2	79						0	0	0		0	0	0		0
Prostate	0		97						44	0				0				0	
Sem Vesicle-R	0		2						0	0				0				0	
Sem Vesicle-L	0		2						98	0				0				0	
Parietal Lobe A R			194	29	56,036								1	0	0	0	0	0	0
Parietal Lobe B R			188	145	8,452								0	0	0	0	0	0	0
Parietal Lobe C R	5,555	37	740	67	31,244	0	108,626	912,277	21,769	0	0	0	0	0	0	0	0	0	0
Parietal Lobe A L			26	33	9,048								0	0	0	0	0	0	0
Parietal Lobe B L			173	578	147,885								0	0	0	0	0	0	0
Parietal Lobe C L	74,584	19	145	20	178,499	0	72,386	132,292	36,991	0	0	0	0	0	0	0	0	0	0
Frontal Lobe A-R			72	46	3,313								0	0	0	0	0	0	0
Frontal Lobe B-R			72	141	5,034								0	0	0	0	0	0	0
Frontal Lobe C-R	31	4	301	103	52,756	0	262,090	99,241	5,584	0	0	0	0	0	0	0	0	0	0
Frontal Lobe A-L			176	0	1,331								0	0	0	0	0	0	0
Frontal Lobe B-L			358	68	8,070								0	0	0	0	0	0	0
Frontal Lobe C-L	199	2	67	495	33,849	0	89,889	1,532,160	8,664	0	0	0	0	0	0	0	0	0	0
Occipital Lobe A-R			896	36	146,664								0	0	0	0	0	0	0
Occipital Lobe B-R			123	155	64,935								0	0	0	0	0	0	0
Occipital Lobe C-R	728	4	46	184	341,869	0	18,871	74,035	19,033	0	0	0	0	0	0	0	0	0	0
Occipital Lobe A-L			80	306	93,531								0	0	0	0	0	0	0
Occipital Lobe B-L			733	120	587,352								0	0	0	0	0	0	0
Occipital Lobe C-L	1,364	28	703	182	204,944	0	94,953	10,139,246	21,020	0	0	0	0	0	0	0	0	0	0
Temporal Lobe A-R			1,422	120	6,987								0	0	0	0	0	0	0
Temporal Lobe B-R			1,199	75	456,638								0	0	0	0	0	0	0
Temporal Lobe C-R	1,600	43	597	72	4,652	0	55,436	24,843	5,364	0	0	0	0	0	0	0	0	0	0
Temporal Lobe A-L			1,101	68	15,155								0	0	0	0	0	0	0
Temporal Lobe B-L			506	85	131,458								0	0	0	0	0	0	0
Temporal Lobe C-L	710	83	386	136	49,908	0	5,457	5,046,703	34,405	0	0	0	0	0	0	0	0	0	0
Cerebellum	139	5	2	45	526	0	3,703	24,604	1,521	0	0	0	0	0	0	0	0	0	0
MSG	0	74	38	0	464	0	0	0	0	0	0	0	0	0	0	0	0	0	0
PSG	0	273	865	51	328	0	0	0	0	0	0	0	0	0	0	0	0	0	0
Ventricle-R	165	203	508	30	281	0	0	8,179	31	0	0	0	0	0	0	0	0	0	0
Ventricle-L	94	114	405	49	97	0	0	1,512	99	0	0	0	0	0	0	0	0	0	0
Lung-R Cranial	144	58	1,410	462	1,221	0	0	96	0	0	0	0	0	0	0	0	0	0	0
Lung-L Cranial	283	77	1,573	678	1,419	0	0	105	0	0	0	0	0	0	0	0	0	0	0
Lung-R Middle	132	221	1,531	1,040	110	27	0	28	0	0	0	0	0	0	0	0	0	0	0
Lung-L Middle	341	373	1,367	393	950	0	0	82	0	0	0	0	0	0	0	0	0	0	0
Lung-R Caudal	83	1,316	1,985	786	1,891	0	0	174	0	0	0	0	0	0	0	0	0	0	0
Lung-L Caudal	34	21	1,140	509	917	0	0	66	0	0	0	0	0	0	0	0	0	0	0
Lung-Accessory	49	1,165	959	1,279	1,040	0	0	88	0	0	0	0	0	0	0	0	0	0	0
Mes LN	0	355	395	162	591	0	146	61	20	0	0	0	0	0	0	0	0	0	0
Stomach	0	9	97	0	27	0	31	83	4	0	0	0	0	0	0	0	0	0	0
Duodenum	2	838	49	0	986	0	0	154	3	0	0	0	0	0	0	0	0	0	0
Jejunum	0	0	16	0	13	0	57	110	2	0	0	0	0	0	0	0	0	0	0
Ileum	0	35	414	0	90	0	46	164	8	0	0	0	0	0	0	0	0	0	0
Colon	35	31	17	0	34	0	0	371	10	0	0	0	0	0	0	0	0	0	0
Cord	0	0	3	0	79	0	0	44	9	0	0	0	0	0	0	0	0	0	0
Decidua	0	0	0	278	24	18	66	2	0	0	0	0	0	0	0	0	0	0	0
Membranes	3	0	0	0	100	0	166	752	0	0	0	0	0	0	0	0	1	0	0
Placenta	56	832	66	125	163	0	43	120	0	0	0	0	0	0	0	0	0	0	0
Muscle	3	3	6	30	85	0	66	457	111	1,200	0	0	0	0	0	0	0	0	0
Plasma (EDTA)-Dam*	0	0	0	0	0	0	0	0	0	0	0	0	0	0	0	0	0	0	0
Oral Swab-Dam*	14,568	5,616	0	0	0	0	0	0	0	0	0	132,204	0	0	0	0	6,048	0	0
Plasma (EDTA)-Fetus*	272	218	6,268	0	290	0	0	6,160	0	0	0	0	0	0	0	0	0	0	0
Urine-Fetus*	41,162	89,442	343,200	0	118,440		1,076	8,340	0	0	0	0	0	0	0	0	0	0	0
Bone Marrow	0	39	0	0	6	0	0	0	1	0	0	0	2	0	0	0	0	0	0
Diaphragm	57	10	112	94	1	0	0	400	0	0	0	0	0	0	0	0	0	0	0
Amniotic Fluid	3,502	9,896	357,112	18,432	69,680	0	0	107,629	0	0	0	0	0	0	0	0	0	0	0
CSF	0	0	0	0	2,228,120		1,209	0	98,800		0	0	0	0	0	0	0	0	0
**RhCMV copies/μg DNA**	**No Sample**		**0**	**<10**	**10^1 - 10^2**	**10^2 - 10^3**	**10^3 - 10^4**	**10^4 - 10^5**	**10^5 - 10^6**	**>10^6**									

Supplemental Figure 2: Rh108-deficient 68-1 RhCMV does not spread in fetal rhesus macaques. (A) Summary of viral genome copy numbers determined in fetal tissues after direct inoculation with indicated viruses. All surviving fetuses were assessed in the late third trimester. Samples for each of the tissues or fluids were collected and processed for qPCR to determine genome copy numbers. The color code refers to the number of DNA copies normalized per 1 µg of DNA (or per ml as indicated by *) as shown. Tissues from the frontal, parietal, occipital, and temporal lobes (right and left cerebral hemispheres, one or three samples [A, B, C] per lobe as indicated) were either analyzed as a single sample or by right or left hemisphere and lobe as indicated by merged rows. Ax LN=axillary lymph node, Ing LN=inguinal lymph node, R=right, L=left, MSG=submandibular salivary gland, PSG=parotid salivary gland, Mes LN=mesenteric lymph node, Sem vesicle=seminal vesicle, CSF=cerebrospinal fluid.

Virus	68-1 RhCMV/ΔRh110/SIVrtni pp71 complemented							68-1 RhCMV/ΔRh110/SIVrtni pp71 uncomplemented						
Inocula Titer (*10^6pfu*)	1.0	1.0	1.0	1.0	1.0	1.0	1.0	1.0	1.0	1.0	1.0	1.0	1.0	1.0
**Fetal Tissue**	RM5.1	RM5.2	RM5.3	RM5.4	RM5.5	RM5.6	RM5.7	RM6.1	RM6.2	RM6.3	RM6.4	RM6.5	RM6.6	RM6.7
**Sex**	F	M	M	M	F	M	F	M	M	M	M	F	M	F
Thymus	0	0	0	0	0	0	0	0	0	0	0	0		61
Spleen	0	0	0	0	0	0	0	0	0	0	0	0		92
Liver	0	0	0	0	0	0	0	0	3	0	0	0	5944641	33
Pancreas	0	0	0	41	0	0	0	0	31	0	0	0		0
Skin	0	0	0	0	0	0	0	0	8	0	0	0		0
Gonad-R	704415	0	0	175	0	0	0	0	0	0	0	0		0
Gonad-L	461410	0	0	93	0	0	240	0	10	0	0	0		0
Ax LN-R	0	0	0	0	0	0	1	0	0	0	0	0		0
Ax LN-L	0	0	0	0	0	0	0	0	14	0	0	0		0
Ing LN-R	0	0	0	0	0	0	0	0	207	0	0	0		0
Ing LN-L	0	0	0	0	0	0	0	0	7	0	0	0		0
Adrenal-R	171	0	0	0	0	0	0	0	0	0	0	0		0
Adrenal-L	0	0	0	0	0	0	0	0	13	0	0	0		0
Kidney-R	0	0	0	0	0	0	0	0	12	0	0	0		0
Kidney-L	0	0	0	0	0	0	4	0	0	0	0	0		0
Uterus					0		0		22			0		0
Prostate						0		0	7	0	0			
Sem Vesicle-R						0		0	11	0	0			
Sem Vesicle-L						0		0	7	0	0			
Parietal Lobe A R								0	14174	0	0	0		0
Parietal Lobe B R								0	99325	0	8	0		0
Parietal Lobe C R	0	126	1023	0	0	0	308	0	91819	0	4	0	187163	0
Parietal Lobe A L								0	13194	0	153	0	31897	0
Parietal Lobe B L								0	11916	0	344	0		0
Parietal Lobe C L	0	909	33022	0	0	0	990	0	731	0	366	0	9532	1
Frontal Lobe A-R								0	9893	0	24	0		1
Frontal Lobe B-R								0	4776	0	9	0		0
Frontal Lobe C-R	0	421	908	0	0	0	646	0	6519	0	3	0	29310	0
Frontal Lobe A-L								0	4597	0	12	5		0
Frontal Lobe B-L								0	2474	0	150	0		5
Frontal Lobe C-L	0	3484	701209	0	0	0	541	0	2584	0	429	0		0
Occipital Lobe A-R								0	2352	0	13	0		0
Occipital Lobe B-R								0	11044	0	1	0	190371	0
Occipital Lobe C-R	0	534	2770	0	0	0	126	0	11587	0	12	0	254223	0
Occipital Lobe A-L								0	807	0	332	0		0
Occipital Lobe B-L								0	10122	0	504	0		0
Occipital Lobe C-L	0	1813	24524	0	0	0	683	0	4845	0	173	0		0
Temporal Lobe A-R								0	138248	0	13	0		0
Temporal Lobe B-R								0	23730	0	0	0		0
Temporal Lobe C-R	0	337	2711	0	0	0	436	0	23419	0	1	0		0
Temporal Lobe A-L								0	569	0	33	0		0
Temporal Lobe B-L								0	4639	0	3	0		0
Temporal Lobe C-L	0	822	382	0	0	0	43	0	5341	0	0	1		2
Cerebellum	0	0	219	0	0	0	60	0	472	0	30	0	1216	0
MSG	0	0	0	0	0	0	0	0	0	0	0	0	137600	0
PSG	0	0	0	0	0	0	0	0	0	0	0	0		0
Ventricle-R	0	0	0	0	0	0	0	0	5	0	0	0		0
Ventricle-L	0	0	0	0	0	0	0	0	4	0	0	0		0
Lung-R Cranial	0	0	0	0	0	0	0	0	9	0	0	0		0
Lung-L Cranial	0	0	0	0	0	0	0		0	0	0	1		0
Lung-R Middle	0	0	0	0	0	0	0		0	0	0	0		0
Lung-L Middle	0	0	0	0	0	0	0		0	0	0	0		0
Lung-R Caudal	0	0	0	0	0	0	0		6	0	0	0		0
Lung-L Caudal	0	0	0	0	0	0	0		10	0	0	0		0
Lung-Accessory	0	0	0	0	0	0	0		77	0	0	0		0
Mes LN	0	0	0	0	0	0	0		274	0	0	0		0
Stomach	0	0	0	0	0	0	0		9	0	0	0		11
Duodenum	0	0	0	0	0	0	0		152	0	0	0		80
Jejunum	0	0	0	0	0	0	0		8	0	0	0		0
Ileum	0	0	0	0	0	0	0		0	0	0	0		0
Colon	0	0	0	0	0	0	0		12	0	0	1	58945	0
Cord	0	0	0	0	0	0	0		0	0	0	0	9	2
Decidua	0	0	0	0	0	0	0		0	0	0	0	44359	0
Membranes	0	0	0	0	0	0	0		0	0	0	0	11600	0
Placenta	0	0	0	0	0	0	0		0	0	0	0		0
Muscle	0	0	0	0	0	0	0		9	0	0	0		0
Plasma (EDTA)-Dam*	0	0	0	0	0	0	0		0	0	0	0		
Oral Swab-Dam*	325888	0	0	0	0	0	0	14880	0	0	4812	0		
Plasma (EDTA)-Fetus*	0	0	0	0	0	0	0	0	0					
Urine-Fetus*				29562	0	0		0	0	0	0	0		470
Bone Marrow	0	0	0	0	0	0	0			0	0	0		0
Diaphragm	3762	124	0	68	0	0	0		52	0	0	0		213
Amniotic Fluid	0	0	0	0	0	0	0	0	0	0	0		13808241	0
CSF	0	0	0	0	0	0	2800	0	0	0	0	0		0

RhCMV copies/µg DNA	No Sample	0	<10	10^1 - 10^2	10^2 - 10^3	10^3 - 10^4	10^4 - 10^5	10^5 - 10^6	>10^6

<u>Supplemental Figure 2 (continued):</u> (B) Genome copy numbers determined in fetal tissues upon direct inoculation with 68-1 RhCMV/ΔRh110/SIVrtni either recovered from pp71-complementing cells or grown without complementation (3) as indicated. Fetuses were either assessed as in (A) in the late third trimester or earlier upon imminent demise (RM6.2 60 dpi) or fetal death (RM6.6: 18 dpi). The two cohorts were combined for comparison with the cohorts shown in Fig. S1A.

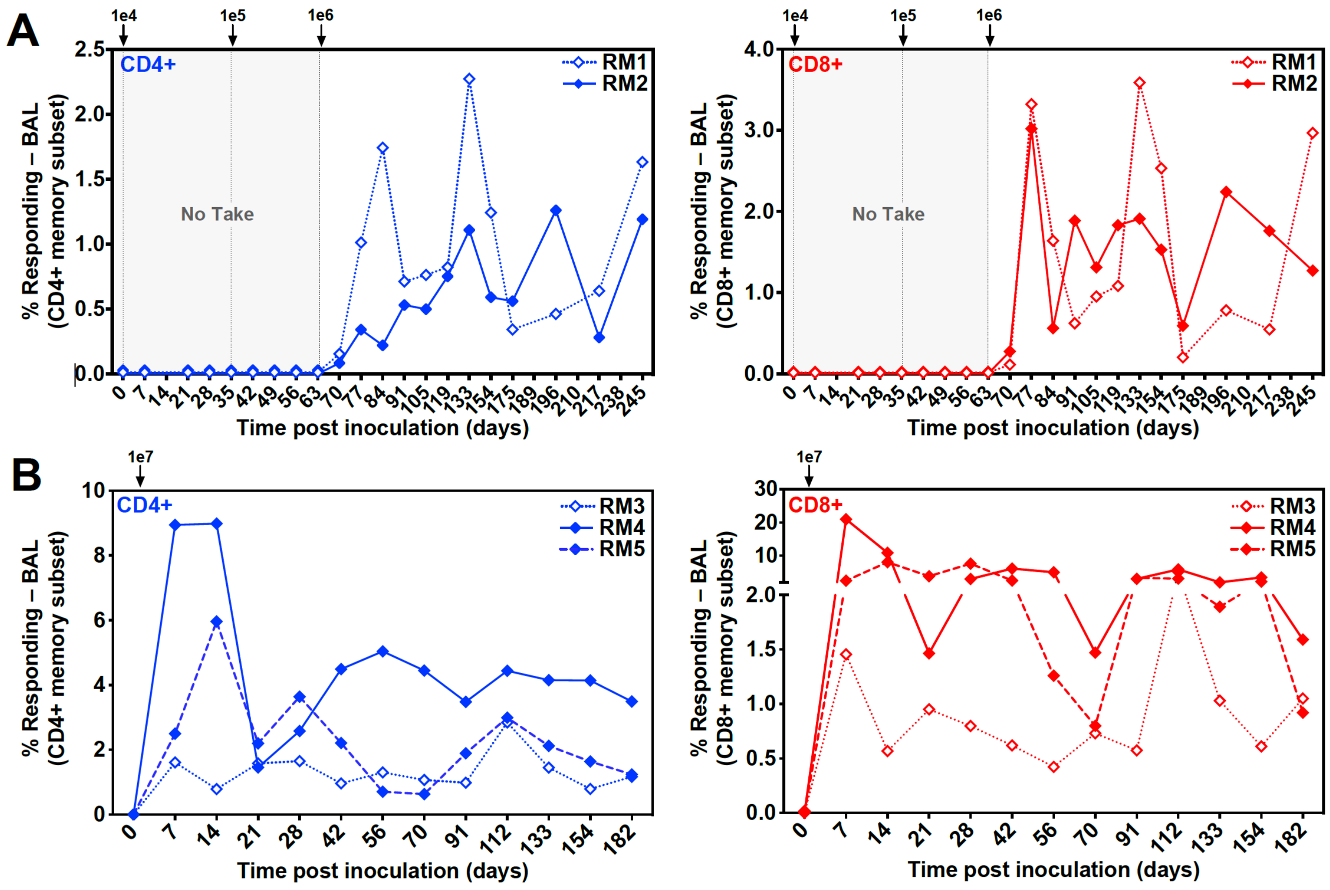

<u>Supplemental Figure 3</u>: SIV-specific CD4⁺ and CD8⁺ T cell responses in BAL. (**A,B**) SIV Rev/Tat/Nef-specific T cell responses were measured in BAL fluid by ICS in RMs 1-5 as described in **Fig. 3A** and **3B**, respectively.

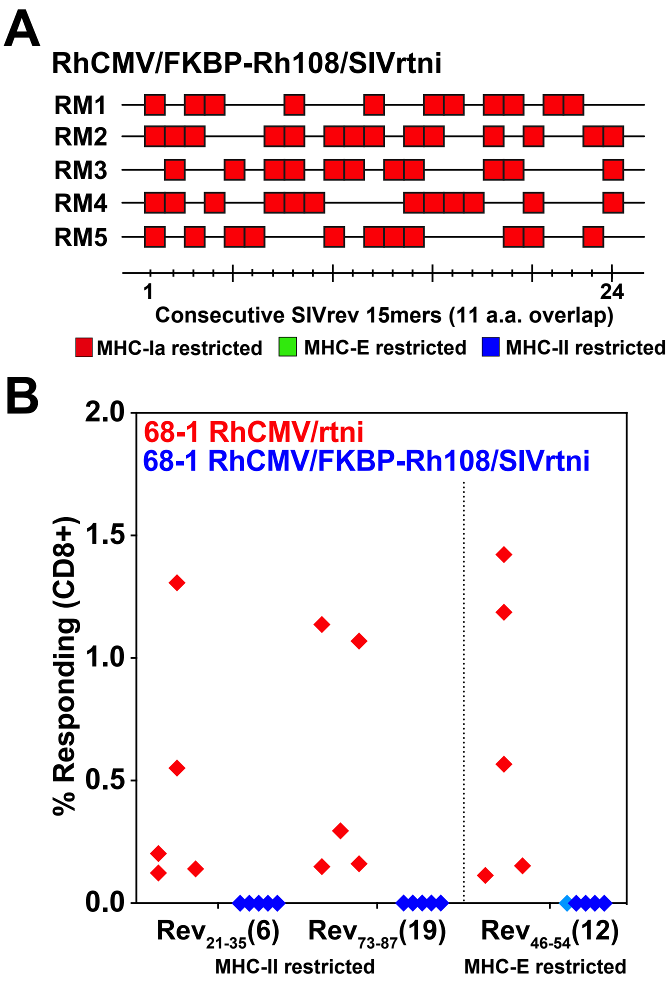

<u>Supplemental Figure 4</u>: Epitope recognition and MHC-restriction analysis of SIV Rev-specific CD8⁺ T cell responses in RM vaccinated with 68-1 RhCMV/FKBP-Rh108/SIVrtni. (**A**) Plateau phase peripheral blood CD8⁺ T cells from the 5 RMs inoculated with 68-1 RhCMV/ FKBP-Rh108/SIVrtni vectors shown in **Figs. 3 and S3** (RMs 1-5) were assessed by flow cytometric ICS assay (TNF-α and/or IFN-γ readout) for responses to each of 25 consecutive (11 amino acid overlapping) 15mer SIV Rev peptides with any above threshold response (> 0.05% after background subtraction) indicated by a box. Boxes are colored to reflect MHC restriction based on the ability to inhibit the response with the MHC-E blocking peptide VL9, the MHC-II blocking mAb G46.6, and/or the pan-MHC-I blocking mAb W6/32 (see Methods). (**B**) CD8+ T cell responses to the indicated individual MHC-E- and MHC-II-restricted SIV Rev supertope peptides (22) were determined by ICS in peripheral blood of 5 RM inoculated with parental 68-1 RhCMV/SIVrtni or the 5 RM from (A) at plateau phase (68-1: 378 – 455 dpi, FKBP: 825 – 855 dpi).

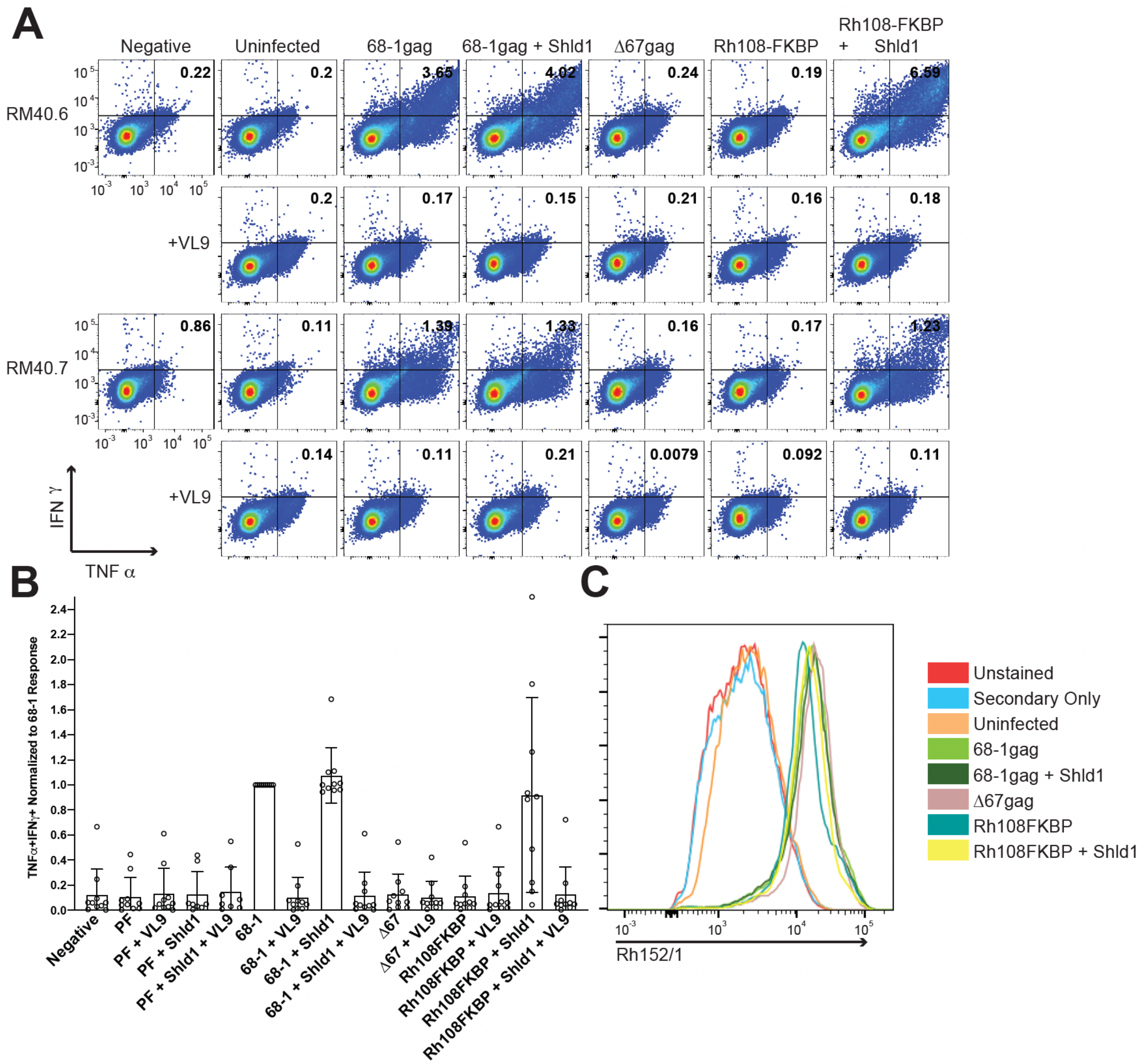

<u>Supplemental Figure 5</u>: Rh108-expression is required for T cell recognition of RhCMV-infected fibroblasts. (**A**) 68-1 RhCMV/SIVgag or derivative recombinants engineered to lack Rh67 or express FKBP-Rh108 were used to infect rhesus fibroblasts at an MOI of 3 in the presence of absence of 1 µM *Shield-1*. After 48 hours, these cells, or uninfected control cells, were co-cultured with CD8+ T cells obtained from 68-1 RhCMV/SIV-vaccinated RMs followed by flow cytometric ICS analysis for IFN-γ and TNF expression. Where indicated, VL9 peptide for blocking MHC-E was added to the assay. The percentage of IFNγ- and TNFα-positive CD8+ T cells are shown in each profile. (**B**) Average frequencies (+/- SEM) of CD8+ T cell responses determined as in (A) from 10 ICS assays using T cells from 4 RM (3 repeats of RMs 40.6 and 40.7 and 2 repeats of RMs 40.8 and 40.9). Frequencies were normalized to T cell responses to 68-1 RhCMV. (**C**) Comparable infection of fibroblasts with the indicated constructs was demonstrated at 48 hours post-infection using a monoclonal antibody recognizing the RhCMV glycoprotein gp68 (Rh152/Rh151) (2).

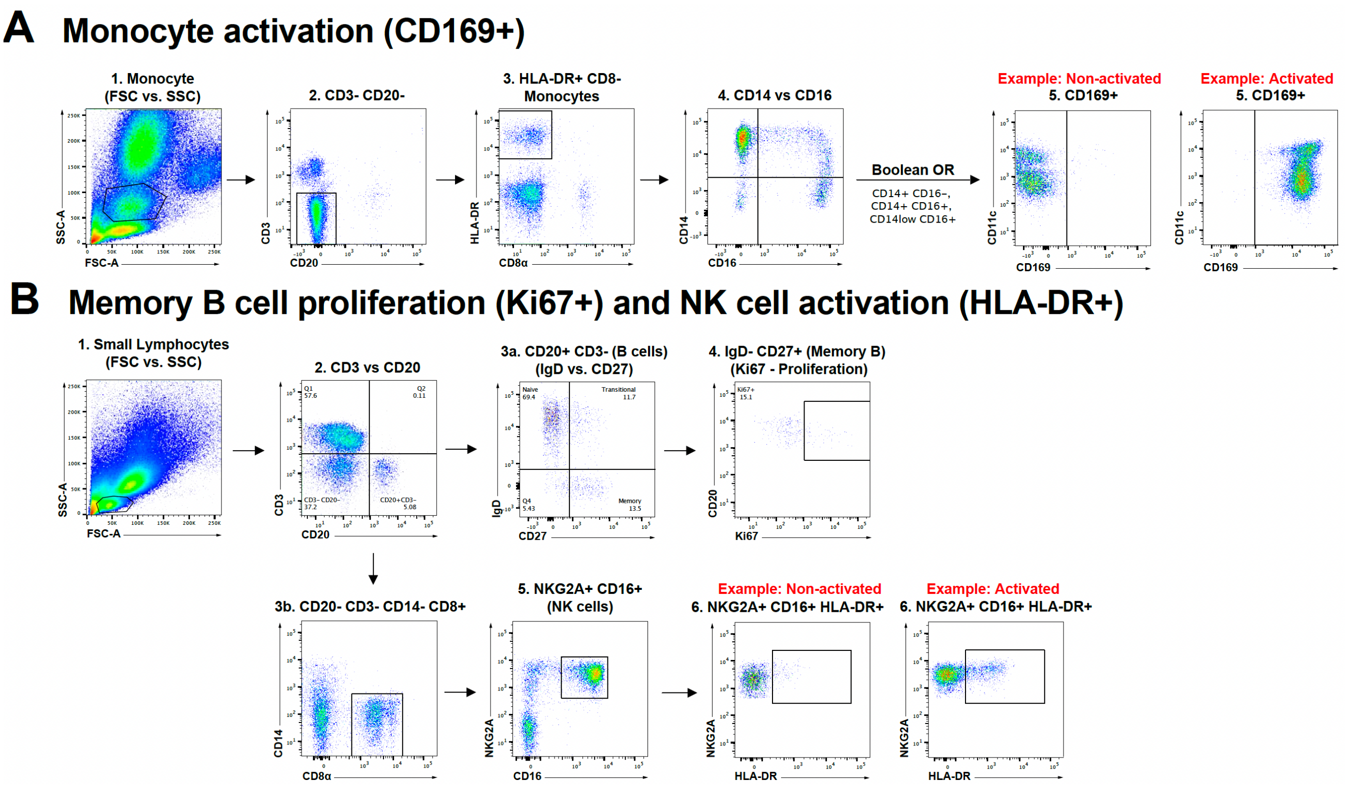

<u>Supplemental Figure 6</u>: Gating hierarchies for flow cytometric analysis of monocyte, NK cell and B cell responses to vaccination. The figure illustrates the progressive gating used to assess monocyte activation, memory B cell proliferation and NK cell activation, based on expression of CD169, Ki-67, and HLA-DR, respectively. The black lines/boxes indicate the progressively gated populations until the final population is quantitatively assessed for activation/proliferation Ag expression based on expression levels indicated by the marker lines.

References

1. Chang WL, Tarantal AF, Zhou SS, Borowsky AD, and Barry PA. A recombinant rhesus cytomegalovirus expressing enhanced green fluorescent protein retains the wild-type phenotype and pathogenicity in fetal macaques. *J Virol.* 2002;76(18):9493-504.
2. Malouli D, Nakayasu ES, Viswanathan K, Camp DG, 2nd, Chang WL, Barry PA, et al. Reevaluation of the Coding Potential and Proteomic Analysis of the BAC-Derived Rhesus Cytomegalovirus Strain 68-1. *J Virol.* 2012;86(17):8959-73.
3. Marshall EE, Malouli D, Hansen SG, Gilbride RM, Hughes CM, Ventura AB, et al. Enhancing safety of cytomegalovirus-based vaccine vectors by engaging host intrinsic immunity. *Sci Transl Med.* 2019;11(501).
4. Chang WL, Kirchoff V, Pari GS, and Barry PA. Replication of rhesus cytomegalovirus in life-expanded rhesus fibroblasts expressing human telomerase. *J Virol Methods.* 2002;104(2):135-46.
5. Oxford KL, Strelow L, Yue Y, Chang WL, Schmidt KA, Diamond DJ, et al. Open reading frames carried on UL/b' are implicated in shedding and horizontal transmission of rhesus cytomegalovirus in rhesus monkeys. *J Virol.* 2011;85(10):5105-14.
6. Taher H, Mahyari E, Kreklywich C, Uebelhoer LS, McArdle MR, Mostrom MJ, et al. In vitro and in vivo characterization of a recombinant rhesus cytomegalovirus containing a complete genome. *PLoS Pathog.* 2020;16(11):e1008666.
7. Rivailler P, Kaur A, Johnson RP, and Wang F. Genomic sequence of rhesus cytomegalovirus 180.92: insights into the coding potential of rhesus cytomegalovirus. *J Virol.* 2006;80(8):4179-82.
8. Assaf BT, Mansfield KG, Strelow L, Westmoreland SV, Barry PA, and Kaur A. Limited dissemination and shedding of the UL128 complex-intact, UL/b'-defective rhesus cytomegalovirus strain 180.92. *J Virol.* 2014;88(16):9310-20.
9. Bialas KM, Tanaka T, Tran D, Varner V, Cisneros De La Rosa E, Chiuppesi F, et al. Maternal CD4+ T cells protect against severe congenital cytomegalovirus disease in a novel nonhuman primate model of placental cytomegalovirus transmission. *Proc Natl Acad Sci U S A.* 2015;112(44):13645-50.
10. Hansen SG, Vieville C, Whizin N, Coyne-Johnson L, Siess DC, Drummond DD, et al. Effector memory T cell responses are associated with protection of rhesus monkeys from mucosal simian immunodeficiency virus challenge. *Nat Med.* 2009;15(3):293-9.
11. Hansen SG, Vieville C, Whizin N, Coyne-Johnson L, Siess DC, Drummond DD, et al. Addendum: Effector memory T cell responses are associated with protection of rhesus monkeys from mucosal simian immunodeficiency virus challenge. *Nature Medicine.* 2011;17:1692.
12. Perng YC, Qian Z, Fehr AR, Xuan B, and Yu D. The human cytomegalovirus gene UL79 is required for the accumulation of late viral transcripts. *J Virol.* 2011;85(10):4841-52.
13. Banaszynski LA, Chen LC, Maynard-Smith LA, Ooi AG, and Wandless TJ. A rapid, reversible, and tunable method to regulate protein function in living cells using synthetic small molecules. *Cell.* 2006;126(5):995-1004.
14. Yu D, Ellis HM, Lee EC, Jenkins NA, Copeland NG, and Court DL. An efficient recombination system for chromosome engineering in Escherichia coli. *Proc Natl Acad Sci U S A.* 2000;97(11):5978-83.

15. Hel Z, Tryniszewska E, Tsai WP, Johnson JM, Harrod R, Fullen J, et al. Design and in vivo immunogenicity of a polyvalent vaccine based on SIVmac regulatory genes. *DNA Cell Biol.* 2002;21(9):619-26.
16. Hansen SG, Ford JC, Lewis MS, Ventura AB, Hughes CM, Coyne-Johnson L, et al. Profound early control of highly pathogenic SIV by an effector memory T-cell vaccine. *Nature.* 2011;473(7348):523-7.
17. Yu D, Smith GA, Enquist LW, and Shenk T. Construction of a self-excisable bacterial artificial chromosome containing the human cytomegalovirus genome and mutagenesis of the diploid TRL/IRL13 gene. *J Virol.* 2002;76(5):2316-28.
18. Caposio P, van den Worm S, Crawford L, Perez W, Kreklywich C, Gilbride RM, et al. Characterization of a live-attenuated HCMV-based vaccine platform. *Sci Rep.* 2019;9(1):19236.
19. Malouli D, Hansen SG, Nakayasu ES, Marshall EE, Hughes CM, Ventura AB, et al. Cytomegalovirus pp65 limits dissemination but is dispensable for persistence. *J Clin Invest.* 2014;124(5):1928-44.
20. Malouli D, Hansen SG, Hancock MH, Hughes CM, Ford JC, Gilbride RM, et al. Cytomegaloviral determinants of CD8(+) T cell programming and RhCMV/SIV vaccine efficacy. *Sci Immunol.* 2021;6(57).
21. Sequar G, Britt WJ, Lakeman FD, Lockridge KM, Tarara RP, Canfield DR, et al. Experimental coinfection of rhesus macaques with rhesus cytomegalovirus and simian immunodeficiency virus: pathogenesis. *J Virol.* 2002;76(15):7661-71.
22. Hansen SG, Hancock MH, Malouli D, Marshall EE, Hughes CM, Randall KT, et al. Myeloid cell tropism enables MHC-E-restricted CD8(+) T cell priming and vaccine efficacy by the RhCMV/SIV vaccine. *Sci Immunol.* 2022;7(72):eabn9301.
23. Hansen SG, Marshall EE, Malouli D, Ventura AB, Hughes CM, Ainslie E, et al. A live-attenuated RhCMV/SIV vaccine shows long-term efficacy against heterologous SIV challenge. *Sci Transl Med.* 2019;11(501).
24. Hansen SG, Piatak M, Jr., Ventura AB, Hughes CM, Gilbride RM, Ford JC, et al. Immune clearance of highly pathogenic SIV infection. *Nature.* 2013;502(7469):100-4.
25. Nelson CS, Cruz DV, Tran D, Bialas KM, Stamper L, Wu H, et al. Preexisting antibodies can protect against congenital cytomegalovirus infection in monkeys. *JCI Insight.* 2017;2(13).
26. Barry PA, Lockridge KM, Salamat S, Tinling SP, Yue Y, Zhou SS, et al. Nonhuman primate models of intrauterine cytomegalovirus infection. *ILAR J.* 2006;47(1):49-64.
27. Tarantal AF, Salamat MS, Britt WJ, Luciw PA, Hendrickx AG, and Barry PA. Neuropathogenesis induced by rhesus cytomegalovirus in fetal rhesus monkeys (Macaca mulatta). *J Infect Dis.* 1998;177(2):446-50.
28. Tarantal AF. *The Laboratory Primate*. Cambridge: Elsevier Academic Press; 2005:317-5.
29. Verweij MC, Hansen SG, Iyer R, John N, Malouli D, Morrow D, et al. Modulation of MHC-E transport by viral decoy ligands is required for RhCMV/SIV vaccine efficacy. *Science.* 2021;372(6541).
30. Hansen SG, Wu HL, Burwitz BJ, Hughes CM, Hammond KB, Ventura AB, et al. Broadly targeted CD8(+) T cell responses restricted by major histocompatibility complex E. *Science.* 2016;351(6274):714-20.
31. Hansen SG, Sacha JB, Hughes CM, Ford JC, Burwitz BJ, Scholz I, et al. Cytomegalovirus vectors violate CD8+ T cell epitope recognition paradigms. *Science.* 2013;340(6135):1237874.

32. Hansen SG, Zak DE, Xu G, Ford JC, Marshall EE, Malouli D, et al. Prevention of tuberculosis in rhesus macaques by a cytomegalovirus-based vaccine. *Nat Med.* 2018;24(2):130-43.
33. Benjamini Y. Controlling the false discovery rate: a practical and powerful approach to multiple testing. *J R Stastist Soc B* 1995;57(1):289-300.
34. Hothorn T, Hornik K, van de Wiel MA, and Zeileis A. Implementing a Class of Permutation Tests: The coin Package. *Journal of Statistical Software.* 2008;28(8):1 - 23.
35. Holm SA. Simple Sequentially Rejective Multiple Test Procedure. *Scandinavian Journal of Statistics.* 1979;6(2):65-70.
36. Dunn OJ. Multiple Comparisons among Means. *Journal of the American Statistical Association.* 1961;56(293):52-64.
